# Histamine H_3_ Receptor Isoforms: Insights from Alternative Splicing to Functional Complexity

**DOI:** 10.3390/biom14070761

**Published:** 2024-06-26

**Authors:** Meichun Gao, Jasper F. Ooms, Rob Leurs, Henry F. Vischer

**Affiliations:** Amsterdam Institute of Molecular and Life Sciences, Division of Medicinal Chemistry, Faculty of Science, Vrije Universiteit Amsterdam, 1081 HZ Amsterdam, The Netherlands; m.c.gao@vu.nl (M.G.); j.f.ooms@vu.nl (J.F.O.); r.leurs@vu.nl (R.L.)

**Keywords:** histamine H_3_ receptor, isoform, signaling, dimerization, RNA sequencing

## Abstract

Alternative splicing significantly enhances the diversity of the G protein-coupled receptor (GPCR) family, including the histamine H_3_ receptor (H_3_R). This post-transcriptional modification generates multiple H_3_R isoforms with potentially distinct pharmacological and physiological profiles. H_3_R is primarily involved in the presynaptic inhibition of neurotransmitter release in the central nervous system. Despite the approval of pitolisant for narcolepsy (Wakix^®^) and daytime sleepiness in adults with obstructive sleep apnea (Ozawade^®^) and ongoing clinical trials for other H_3_R antagonists/inverse agonists, the functional significance of the numerous H_3_R isoforms remains largely enigmatic. Recent publicly available RNA sequencing data have confirmed the expression of multiple H_3_R isoforms in the brain, with some isoforms exhibiting unique tissue-specific distribution patterns hinting at isoform-specific functions and interactions within neural circuits. In this review, we discuss the complexity of H_3_R isoforms with a focus on their potential roles in central nervous system (CNS) function. Comparative analysis across species highlights evolutionary conservation and divergence in H_3_R splicing, suggesting species-specific regulatory mechanisms. Understanding the functionality of H_3_R isoforms is crucial for the development of targeted therapeutics. This knowledge will inform the design of more precise pharmacological interventions, potentially enhancing therapeutic efficacy and reducing adverse effects in the treatment of neurological and psychiatric disorders.

## 1. Alternative Splicing to Increase GPCRome Diversity

Alternative splicing is the post-transcriptional processing of precursor messenger RNA (pre-mRNA) in which non-coding introns are excised and the protein-coding exons can be joined in different combinations to form mature mRNA transcripts that are subsequently translated into distinct protein isoforms. Alternative splicing involves various mechanisms such as exon skipping, mutually exclusive exons, alternative splice site selection, and intron retention [1]. The splicing pattern is cell- and tissue-specific [2], which enables the precise regulation of physiological processes and adds complexity and versatility to cellular functions. In some cases, specific isoforms generated by alternative splicing may play a significant role in the pathogenesis or progression of diseases such as cancer and neurological disorders [3].

G protein-coupled receptors (GPCR) are the largest family of cell surface receptors with a conserved seven transmembrane (7TM) structure that are involved in cellular communication by transducing a specific extracellular (chemical) signal into an intracellular response. GPCR proteins are involved in the regulation of virtually all physiological processes in the body, and, with 34% of the US Food and Drugs Administration (FDA)-approved therapeutics acting on this receptor family, are one of the most important drug targets [4]. In a recent analysis of RNA sequencing data (version 7) from the Genotype-Tissue Expression (GTEx) consortium that included 53 tissues from 714 donors [5], it was discovered that 42% of the 111 GPCRs with FDA-approved drugs express multiple isoform mRNAs with distinct tissue-specific (co-) expression patterns [6]. GPCR alternative splicing contributes to the diversity of GPCR signaling, including altered ligand binding affinities, signaling properties, and cellular response [3]. Consequently, drug responsiveness could be affected due to the combinatorial expression of GPCR isoforms in vivo [6].

The histamine H_3_ receptor (H_3_R) is primarily involved in the presynaptic inhibitory regulation of neurotransmitter release in the central nervous system (CNS) and is consequently considered as potential therapeutic target for various neurological and psychiatric diseases [7,8,9]. In 2016, the H_3_R antagonist/inverse agonist pitolisant (Wakix^®^) was approved as a treatment for narcolepsy, and, in 2019, as Ozawade^®^ for excessive daytime sleepiness in individuals with sleep apnea [10,11,12], as well as several other H_3_R antagonists/inverse agonists in (pre-)clinical trials [8]. Importantly, 20 different human H_3_R splice variants have been reported in peer-reviewed and patent literature soon after the initial cloning of this receptor approximately 2.5 decades ago [13,14,15,16,17,18,19,20]. Yet, the physiological functional significance of H_3_R isoforms remains still enigmatic to date as only a few of them have been pharmacologically characterized in heterologous expression systems and the lack of selective tools has prevented analysis of their spatial distribution in great detail. Consequently, in in vivo and ex vivo studies, it is not known which endogenous H_3_R isoforms contribute to the observed responses on the protein level in specific brain areas (see Section 4.1, Section 4.2, Section 4.3 and Section 4.4), which hampers in-depth understanding of their (patho-) physiological function. Moreover, initial drug discovery and lead optimization programs have mostly been focused on the H_3_R-445 reference variant (vide infra) and/or do not discriminate between isoforms despite their pharmacological differences [11].

Interestingly, the RNA sequencing data provided by the GTEx consortium identified the expression of two human H_3_R splice variants in various brain regions [21], whereas a more recent study that encompassed RNA-sequencing data in 48 human tissues from the Gene Expression Omnibus (https://tools.hornlab.org/Splice-O-Mat/; accessed on 23 April 2024) revealed the differential expression of 7 human H_3_R isoform mRNAs in various brain regions, of which 4 have not been identified before (see Section 3) [22].

In this review, we highlight the critical need to understand the diverse expression and functionality of H_3_R isoforms for drug discovery programs, and to explore their potential as targets for more effective therapeutic interventions in neurological and psychiatric disorders. By exploring the evolutionary conservation and divergence of H_3_R splicing across species and analyzing recent RNA sequencing data, this review aims to shed some light on possible isoform-specific functions and interactions within neural circuits that have so far been reported in literature (mostly) without knowing which isoform protein is actually involved.

## 2. Histamine H_3_ Receptor (H_3_R) and Its Isoforms: Within and across Species

In the CNS, histamine is produced by neurons located in the tuberomammillary nucleus (TMN) situated in the posterior hypothalamus. These histaminergic neurons send projections throughout the brain, playing a crucial role in the regulation of arousal, wakefulness, and feeding by activating postsynaptic histamine receptors (H_1_R, H_2_R, and/or H_3_R) on glutamatergic, cholinergic and GABAergic neurons [23,24,25,26]. While the roles of H_1_R, H_2_R, and H_3_R are well-documented, the function of the H_4_R in the CNS remains less understood. Nonetheless, evidence supports the expression of H_4_R in brain tissues from both rats and patients with Parkinson’s disease (PD) and amyoptrophic lateral sclerosis (ALS), suggesting potential but yet to be fully elucidated roles [27,28,29]. In addition, H_4_R-deficient mice revealed that H_4_R influences various neurophysiological processes, such as locomotor activity, nociception, anxiety, and feeding behavior [30]. The H_3_R was demonstrated for the first time in 1983 as a presynaptic autoreceptor that inhibited histamine release from depolarized rat cerebral cortex slices due to a distinct pharmacological profile as compared to the earlier identified histamine H_1_ and H_2_ receptors (H_1_R and H_2_R) [31]. Thereafter, this new histamine receptor was shown to be involved in the regulation of a number of other neurotransmitters, i.e., as a heteroreceptor [25].

**Figure 1:**
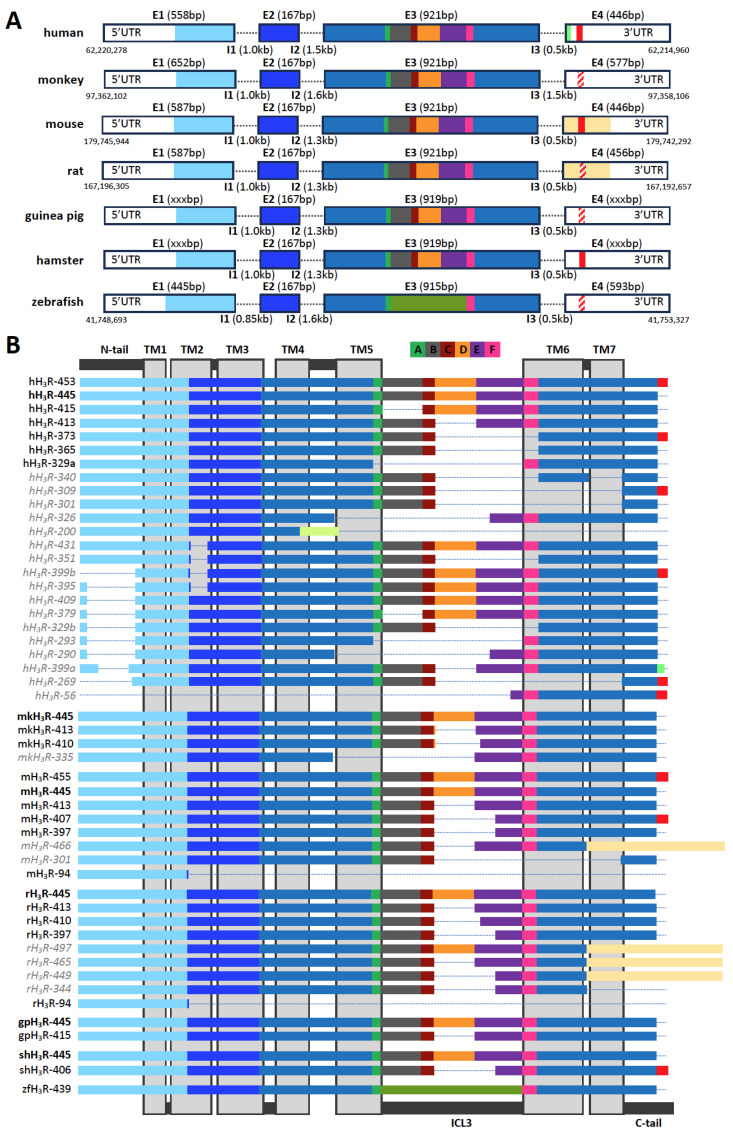
*HRH3* gene structure and identified H_3_R isoforms in seven species. (**A**) Genome sequences of human chromosome 20 (NC_000020.11), monkey chromosome 10 (NC_041763.1), mouse chromosome 2 (NC_000068.8), rat chromosome 3 (NC_086021.1), guinea pig (NW_026947488.1), Siberian hamster (JANBXA010000429), and zebrafish chromosome 7 (NC_007118.7) were retrieved from GenBank (https://www.ncbi.nlm.nih.gov/nuccore/; accessed on 1 April 2024)). Exons 1–4 (E1–E4) and introns 1–3 (I1–I3) are depicted as boxes (on scale) and dashed lines (not on scale), respectively. The colors indicate the H_3_R protein domains that are encoded by the four exons, and, within exon 3, the pseudo intron encoding for ICL3 segments. The six ICL3 segments (A-F) have been defined based on identified human splice variants: A = R^227^–L^233^; B = D^234^–Q^263^; C = K^264^–H^273^; D = R^274^–S^305^; E = S^306^–Q^342^; F = S^343^–D^353^. Hatched red boxes in exon 4 indicate genomic sequences that encode for the extended C-tail but for which transcripts have so far not been detected. (**B**) Schematic protein sequence alignment of human (h), monkey (mk), mouse (m), rat (r), guinea pig (gp), Siberian hamster (sh) and zebra fish (zf) H_3_R isoforms identified so far with exons and different segments (A–F) colored according to the DNA coding sequencing. The snake plot on the background indicates the structural domains of the 7TM GPCRs, with the N- and C-terminal tail, transmembrane (TM) domains, and intracellular loop 3 (ICL3) indicated. The H_3_R isoforms that conserve the prototypical 7TM GPCR folding are indicated in black with reference H_3_R-445 orthologs in bold, whereas isoforms that do not conserve this 7TM folding due to sequence deletions elsewhere in the protein and/or alternative sequences are depicted in grey and italics.

The human H_3_R cDNA was first cloned in 1999 from a thalamus cDNA library as a 445 amino acid-long GPCR (hH_3_R-445) that inhibited cAMP accumulation in transfected cell lines in response to histamine and H_3_R agonists [15]. In addition, an extended hH_3_R (hH_3_R-453) with an eight-amino-acid (^446^KMKKKTCL^453^) extended C-terminal tail and a comparable pharmacological profile was independently identified and reported one year later [16]. The human *HRH3* gene is located on the antisense strand of chromosome 20 q13.33, spanning 5297 nucleotides, and was initially proposed to consist of three exons separated by two introns [17]. However, the 8-amino-acid C-terminal extension in hH_3_R-453 results from a splice donor site just before the stop codon in exon 3 that splices out 604 nucleotides [13,20] and consequently defines intron 3 and exon 4 (Figure 1A), instead of those latter two being the 3′ untranslated region (UTR) in exon 3 [17]. Nonetheless, both three- and four-exon *HRH3* gene structures (accession codes: NM_00732.3 and XM_01702723.2, respectively) have been annotated in the National Center for Biotechnology Information (NCBI) GenBank database (Gene ID: 11255). For several other species, one or more H_3_R isoform cDNAs have been subsequently cloned from the hypothalamus (rat and Siberian hamster), thalamus (monkey), striatum (rat), cerebral cortex (guinea pig), or total brain RNA (rat, mouse, monkey, and zebrafish) [32,33,34,35,36,37,38,39,40,41,42,43]. Except for zebrafish, all these species express the H_3_R-445 isoform (Figure 1B), which is considered to be the canonical H_3_R variant due to its evolutionary conservation but also highest abundancy in the CNS (vide infra). Interestingly, C-terminal extensions comparable with hH_3_R-453 (^446^KMKKKTCL^453^) were only cloned or detected by RNA sequencing in the Siberian hamster (shH_3_R-406; ^398^KMEEKKTRL^406^) and mouse (mH_3_R-407/455; ^398/446^KMEEKKTSSL^407/455^), respectively [34,44]. Both these extensions also originate from the splice donor site that eliminates 604 base pairs (i.e., intron 3) including the canonical stop codon. Sequences encoding this C-terminal extension can be found in the *HRH3* gene of other species, suggesting that they share a similar four-exon structure as humans (Figure 1A). The four-exon gene structure is further supported by 6TM splice variants that have been cloned from rats (rH_3_R-497, rH_3_R-465, and rH_3_R-449) using a 3’UTR reverse primer and RNA sequencing in mice (mH_3_R-466) [41,44], in which 740 and 686 nucleotides, respectively, are spliced-out after the tyrosine at the end of extracellular loop (ECL) 3 (Y^392^ in the canonical rH_3_R-445 and mH_3_R-445) and an alternative 105-amino-acid-long extracellular C-tail is encoded by exon 4 (Figure 1A,B). Interestingly, an alternative extended C-terminal sequence (NVKGP) was found in hH_3_R-399a by RNA sequencing, which is also encoded by exon 4. In all *HRH3* genes, exon 1 encodes for the extracellular N-terminus, TM1, intracellular loop (ICL) 1, and the intracellular half of TM2. Exon 2 encodes for the extracellular half of TM2, ECL1, and TM3. Exon 3 encodes for the remaining helices, loops, and intracellular C-tail of H_3_R, and exon 4 encodes for the extended or alternative C-terminus (Figure 1A,B).

Hitherto, twenty-four human H_3_R isoforms have been identified by cloning and/or RNA sequencing (Table 1) [13,14,15,16,17,18,19,20,21,22,45], with amino acid sequence deletions in the extracellular N-terminal tail, TM2, TM5, TM6, TM7, ECL3, ICL3, or an extension of the C-terminal tail, or a combination of these (Figure 1B). The splice variants with deletions in either the extracellular N-terminus and/or the various TM helices do not conserve the prototypical 7TM GPCR structure and are most likely unable to bind H_3_R ligands and/or trigger intracellular G protein signaling. Indeed, hH_3_R-431 was unable to bind [^125^I]-iodoproxyfan when recombinantly expressed in Chinese hamster ovary K1 subline (CHO-K1) cells, despite a similar subcellular localization as hH_3_R-445 and hH_3_R-365 [13], whereas hH_3_R-301 and hH_3_R-200 were not responsive to histamine in a functional assay in transfected NIH-3T3 cells [20]. The truncated hH_3_R-200 has a deletion of 409 nucleotides in exon 3, resulting in a frameshift that might shorten TM4 with one helical turn and create a unique 30-amino-acid-long extracellular C-terminal tail [20]. In both mice and rats, 94-amino-acid-long truncated isoforms have been identified (mH_3_R-94 and rH_3_R-94) that consist of the N-terminal tail, TM1, ICL1, and a part of TM2 as a consequence of splicing event 4 nucleotides before the end of exon 1 and the consensus splice site in all other mouse and rat isoforms (i.e., CCTC^GTGGgtaa instead of CCTCGTGG^gtaa, respectively), which might be translated as a single transmembrane protein [20,44].

Exon 3 of *HRH3* genes contains multiple cryptic splicing donor and acceptor sequences, allowing the deletion of a pseudo intron and resulting in the existence of numerous isoforms that conserve the 7TM GPCR structure but with a varying length of ICL3 [13,17]. In human H_3_R isoforms (hH_3_R-415, hH_3_R-413, hH_3_R-365/373, and hH_3_R-329a), an individual or combination of six ICL3 sequence segments (A–F) is deleted (Figure 1B). The deletion of segment D in hH_3_R-413 (ΔR^274^-S^305^), starting at nucleotide 822 of the coding sequence, is conserved in other species, including monkeys (mkH_3_R-413), mice (mH_3_R-413), rats (rH_3_R-413), and guinea pigs (gpH_3_R-415). Interestingly, this segment D deletion is extended into a part of segment E in H_3_R isoforms of the monkey (mkH_3_R-410), mouse (mH_3_R-397/407), rat (rH_3_R-410 and rH_3_R-397), and Siberian hamster (shH_3_R-406), whereas, so far only in human isoform hH_3_R-365/373, the deletion of segment D in combination with E and F (ΔR^274^-D^353^) has been encountered. Moreover, also the combined deletion of segments A–E (ΔR^227^-D^353^) in hH_3_R-329a is hitherto unique to humans. Exon 3 pseudo intron deletions are combined with the C-tail extension in hH_3_R-373, mH_3_R-407, and shH_3_R-406, but in humans also with non-7TM deletions in the N-tail/TM1 and/or TM2/TM5/TM6/TM7 (Figure 1B). The H_3_R isoforms with varying lengths for ICL3 and the C-terminal have garnered interest, as these regions are responsible for the selective interaction with intracellular transducer/scaffold proteins such as G proteins and β-arrestins, as reported for other GPCRs [6,46,47].

**Table 1:** H_3_R isoforms from seven species. Alternative names from original reference and GenBank, Uniprot, or the NSTRG, ENST, and ENSMUST RNA sequencing (other) codes from Splice-O-Mat (https://tools.hornlab.org/Splice-O-Mat/; accessed on 23 April 2024), Adult GTEx (https://gtexportal.org/home/transcriptPage; accessed on 23 April 2024), Human Protein Atlas (https://www.proteinatlas.org/about/download; accessed on 23 April 2024), respectively, are provided.

Species	Isoform	Alternative Names	Genbank	Uniprot	RNA-seq	References
Human	453	-	XP_054178891	-		[16,22,45]
	**445**	Isoform 1; GPCR97	NP_009163.2	Q9Y5N1	NSTRG_52062.3; ENST00000340177.9	[13,15,20,22,48,49]
	431	H_3(TM2,431AA)_	-	-		[13]
	415	H_3(Δi3,415AA)_	-	-		[13]
	413	H_3S_	-	-		[17,49]
	409	-	-	-		[14]
	399a	399	XP_016883112.1	-	NSTRG_52062.8	[22]
	399b	399	XM_017027623.1	-		[22]
	395	-	-	-		[14]
	379	-	-	-		[14]
	373	Isoform 4	AF321913	Q8WXZ9	NSTRG_52062.5; ENST00000317393.10	[20,22]
	365	Isoform 2; H_3S_	AF321911	Q8WY01		[13,20,48,49]
	351	-	-	-		[18]
	340	-	-	-		[19]
	329a	H_3(Δi3,329AA)_	-	-		[13,49]
	329b	-	-	-		[14]
	326	H_3(Δi3+TM5,326AA)_	AF346903	-		[13]
	309	Isoform 6	AF346904	Q8NI49		[20]
	301	Isoform 3	AF321912	Q8WY00		[20]
	293	-	-	-		[14]
	290	-	-	-		[14]
	269	-	-	-	NSTRG_52062.7	[22]
	200	Isoform 5	AF346903	Q8NI50	ENST00000611492.1	[20,21]
	56	-	-	-	NSTRG_52062.6	[22]
Monkey (macaca)	**445**	-	AAO63757.1	-		[36]
	413	-	-	-		[35]
	410	-	-	-		[35]
	335	-	-	-		[35]
Mouse	466	-	-	E9Q540	ENSMUST00000163215	[44]
	455	-	-	-	ENSMUST00000165762	[44]
	**445**	Isoform 1	AY044153	P58406	ENSMUST00000056480	[32,40,44]
	413	-	-	E9Q5S3	ENSMUST00000164442	[40,44]
	407	-	-	E9Q7T5	ENSMUST00000165248	[44]
	397	-	-	-		[40]
	301	-	-	E9Q522	ENSMUST00000171736	[44]
	94	-	-	E9PZM9	ENSMUST00000166724	[44]
Rat	497	H_3D_	NP_001257495	Q2VJ18		[41]
	465	H_3E_	NP_001257496	-		[41]
	449	H_3F_	-	-		[41]
	**445**	H_3L_; H_3A_	AY009370	Q9QYN8		[37,38,42]
	413	H_3S_; H_3B_	AY009371	Q541U0		[37,38,42]
	410	isoform 7/8	NP_001257498	-		[38]
	397	H_3C_	-	-		[38,50]
	344	-	BAA88768	-		[51]
	94	H_3(f1)_;H_3T_	-	-		[38]
Guinea pig	**445**	H_3L_	AF267537	Q9JI35		[39]
	415	H_3S_	AF267538	Q9JI36		[39]
Hamster	**445**	Long	AY855070	-		[34]
	406	Short	AY855071	-		[34]
Zebrafish	439	-	ABF71709	-		[43]

## 3. Localization of H_3_R Isoforms in the Central Nervous System

The H_3_R protein is predominantly expressed in the brain as determined by [^3^H]N^α^-methylhistamine binding to membranes isolated from guinea pig tissues [52]. Highest H_3_R expression was observed in the cortex, hypothalamus, striatum, and midbrain (54.5, 42.8, 25.3, and 24.1 fmol/mg membrane protein, respectively). In the periphery, H_3_R is expressed at lower levels in the large intestine, ileum, and pancreas (~5.4 fmol/mg), but is (almost) undetectable in other digestive, respiratory, circulatory, excretory, reproductive, and muscle tissues (<1 fmol/mg).

**Figure 2:**
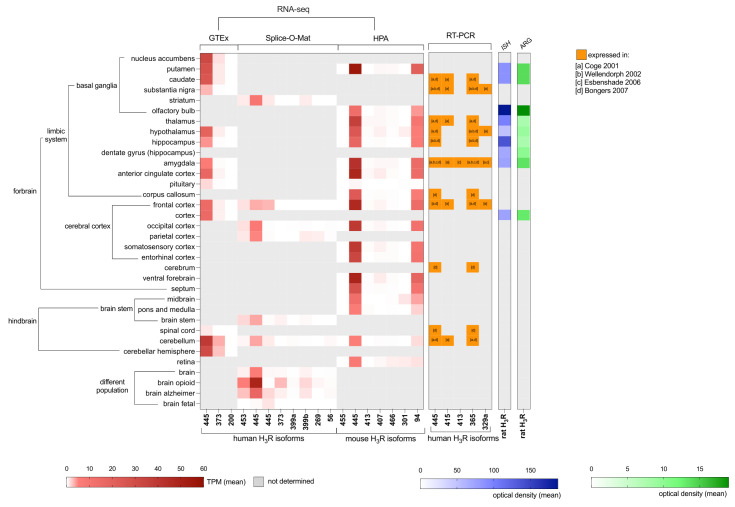
H_3_R isoform expression in the CNS of a human, mouse, and rat. Isoform transcripts in the CNS detected by RNA sequencing and extracted from publicly available Adult GTEx [21], Splice-O-Mat [22], and HPA databases [44], in situ hybridization [54] or RT-PCR [13,20,48,49], and protein level by [^125^I]iodoproxyfan binding [54]. TPM (transcripts per million) represents the relative transcript abundant. Splice-O-Mat: https://tools.hornlab.org/Splice-O-Mat/ (accession date: 23 April 2024); Adult GTEx: https://gtexportal.org/home/transcriptPage (version 8, accession date: 23 April 2024); HPA (mouse): https://www.proteinatlas.org/about/download (version 23.0, accession date: 23 April 2024).

RNA sequencing (RNA-seq) data from the GTEx consortium and Splice-O-Mat that encompass 53 and 48 tissue sample sites in the human body, respectively, confirmed that H_3_R is almost exclusively expressed in the CNS with less than 0.1 TPM detection in other tissues (Figure 2) [21,22]. In both human RNA-seq datasets, but also in mice, the canonical H_3_R-445 is the most abundantly expressed isoform in various brain regions (such as the caudate nucleus, hippocampus, basal ganglia, amygdala, cerebellum, and hypothalamus), which corroborates with the expression profiles by Northern blotting and RT-PCR in earlier studies [13,15,20,48,49]. In contrast to previous studies that reported the abundant expression of hH_3_R-365 next to the canonical hH_3_R-445 in the CNS [13,48,49], both the GTEx and Splice-O-Mat RNA-seq data did not detect the hH_3_R-365 transcripts but instead its C-terminally extended isoform hH_3_R-373 (Figure 1B and Figure 2). Indeed, the study of Cogé and co-workers did not identify extended extended isoform hH_3_R-373 as a reverse oligonucleotide primer based on the stop codon of the canonical hH_3_R-445 was used for cloning [13], whereas the C-terminally extended isoform hH_3_R-373 was cloned by Wellendorph et al. using 3’UTR reverse primers in addition to hH_3_R-365 [20]. Nonetheless, the earlier RT-PCR studies all used primers that flanked ICL3 and consequently did not discriminate between hH_3_R-365 and hH_3_R-373 [13,20,48,49]. The expression of the hH_3_R-453, hH_3_R-399a, hH_3_R-399b, hH_3_R-269, hH_3_R-200, and hH_3_R-56 isoforms was less abundant as compared to hH_3_R-445 according to RNA sequencing. RNA sequencing of a mouse brain revealed the expression of four additional mouse isoforms (mH_3_R-407, mH_3_R-466, mH_3_R-301 and, mH_3_R-94) that were not reported by other techniques before [44]. Surprisingly, the truncated mH_3_R-94 transcript is highly expressed in most brain regions and might perhaps act as single transmembrane anti-chaperone on the expression of other H_3_R isoforms (vide infra), as previously observed for the human H_4_R [53] (Figure 2).

Interestingly, RNA sequencing data from the Splice-O-Mat platform showed that hH_3_R-445, hH_3_R-453, hH_3_R-373, hH_3_R-399a, hH_3_R-399b, hH_3_R-269, and hH_3_R-56 were expressed at higher levels in the brains of individuals with an opioid use disorder (OUD) or Alzheimer’s disease symptoms as compared to the control population (Figure 2). The latter corroborates with the increased H_3_R mRNA levels observed in the prefrontal cortex of female subjects with Alzheimer’s disease [55]. Additionally, the upregulated expression of H_3_R was observed in the dorsolateral prefrontal cortex of individuals with schizophrenia [56], suggesting a potential role for H_3_R dysregulation in neurodegenerative disorders.

The elevated expression of H_3_R in OUD individuals may reflect an adaptive response to chronic opioid exposure. One possible mechanism underlying this upregulation could involve the modulation of histaminergic neurotransmission in response to opioid-induced alterations in neuronal activity. Moreover, the observed upregulation of H_3_R expression in OUD individuals may contribute to the modulation of opioid analgesia and tolerance. Previous studies have demonstrated a synergistic interaction between H_3_R agonists, RAMH, and the opioid fentanyl in animal models, suggesting that increased H_3_R expression could enhance the analgesic effects of opioids [57]. This phenomenon may represent a compensatory mechanism aimed at counteracting the development of tolerance to opioid analgesia, whereby the upregulation of H_3_R serves to potentiate opioid-induced analgesia and mitigate the need for escalating opioid doses. Furthermore, the upregulation of H_3_R expression in OUD individuals highlights the potential therapeutic relevance of targeting H_3_R for the treatment of opioid addiction. Given the role of H_3_R in modulating neurotransmitter release and synaptic plasticity, pharmacological interventions aimed at modulating H_3_R activity could represent a novel approach for managing OUD and reducing opioid-related harms. However, further research is needed to elucidate the precise mechanisms underlying the dysregulation of H_3_R in OUD and to evaluate the therapeutic potential of H_3_R-targeted interventions in the context of opioid addiction.

A comprehensive comparison between autoradiography with the H_3_R-selective radioligand [^125^I]iodoproxyfan and in situ hybridization (ISH) using a ^33^P-labeled riboprobe that recognizes most rH_3_R isoforms in the brains of the same rats (Figure 2) has revealed some discrepancies between receptor binding sites and mRNA expression [54], which can be explained by mRNA being located in perikarya, whereas presynaptic receptor binding occurs on the axon terminals [58]. Comparable distributions of H_3_R in the cerebral cortex, nucleus accumbens, striatum, and substantia nigra were observed in other autoradiography studies using R-[^3^H]-α-methylhistamine and [^125^I]iodophenpropit for rat [59,60], mouse [61], monkey [62] and human [62] cases. Notably, differential expression patterns of rat isoforms rH_3_R-445, rH_3_R-413, and rH_3_R-397 were observed in the dentate gyrus and hippocampal subfields by ISH [42].

In addition to radioligands, antibodies have been used to detect H_3_R expression in the CNS. Detection of its isoforms in the brain can be achieved using specific antibodies that target distinct peptide sequences. Antibodies raised against the RLSRDRKVAK peptide, corresponding to amino acids 349–358 within TM6 of the canonical H_3_R-445 sequence, including the last five amino acids of segment F (Figure 1B), allow for the identification of H_3_R protein expression across various brain regions, including the cerebral cortex, hippocampus, cerebellum, striatum, olfactory tubercle, substantia nigra, and thalamus. This distribution pattern is consistent with previous studies using mRNA and binding site analyses [63]. Additionally, using a different antibody from commercial resources (epitope not disclosed) revealed the H_3_R expression in dopamine D_1_ receptor (D_1_R)-positive interneurons and vesicular glutamate transporter 1 (VGLUT1)-positive corticostriatal output neurons, suggesting a potential functional role through receptor dimerization in neural networks (vide infra) [64]. The presence of the H_3_R protein has been confirmed in the ventral tegmental area and substantia nigra, particularly within dopaminergic neurons [65].

The peptide sequence junctions created by alternative splicing could provide unique epitopes for the generation of isoform-specific antibodies. Indeed, the anti-hH_3_R-329a antibody, raised against a peptide sequence at the junction between TM5 and segment F (CYLNIQ/SFTQR) in hH_3_R-329a (Figure 1), selectively detected this isoform in transfected cells without cross-reacting with hH_3_R-365 and hH_3_R-445 [66]. Similarly, a polyclonal antibody targeting a fourteen amino acid segment in ICL3 of rH_3_R-445 displays specificity for rH_3_R-445 without binding to the shorter rH_3_R-413 or rH_3_R-397 isoforms that lack this sequence in transfected HEK293 cells [67]. However, this antibody was found to also detect the β subunit of ATPase in H_3_R knockout mice. Challenges remain, where the anti-hH_3_R-365 antibody, targeting the peptide sequence including the segment C junction with TM6 (EAMPLH/RKVAKSLAC), has not distinguished between hH_3_R-365 and hH_3_R-445 [66]. Despite these difficulties, the combined use of antibodies such as anti-hH_3_R-365 and anti-hH_3_R-329a has provided valuable insights into the presence and localization of H_3_R isoforms in brain regions like dendrites, the tuberomammillary nucleus (TMN), and substantia nigra (SN) neurons. This understanding contributes to the potential functional diversity of H_3_R isoforms within the brain [66].

## 4. Function and Signaling Transduction of H_3_R

### 4.1. H_3_R Constitutively Activates G_i/o_ Proteins

Pretreatment of rat cerebral cortex membranes with pertussis toxin (PTX) disabled the accumulation of [^35^S]GTPγS in activated heterotrimeric G proteins upon stimulation with the selective H_3_R agonists R^α^-methylhistamine and N^α^-methylhistamine, indicating that endogenous H_3_R signals via Gα_i/o_ proteins [68]. Indeed, systematic evaluation of histamine-induced hH_3_R-445 coupling to chimeric Gα subunits that harbor subtype-specific six-amino-acid C-tail substitutions in engineered human embryonic kidney cells (HEK293) by measuring alkaline phosphatase-fused transforming growth factor-α shedding has confirmed selective coupling to Gα_i1_, Gα_i3_, and Gα_io_ [69]. Moreover, histamine stimulates the activation of heterotrimeric Gα_i1_, Gα_i2_, Gα_i3_, and Gα_io_ by hH_3_R-445 with comparable potencies as measured by the dissociation of these Gα from Gβγ subunits using bioluminescence resonance energy transfer (BRET)-based sensors, suggesting that hH_3_R-445 displays no coupling preference between the PTX-sensitive G_i/o_ proteins [70].

**Figure 3:**
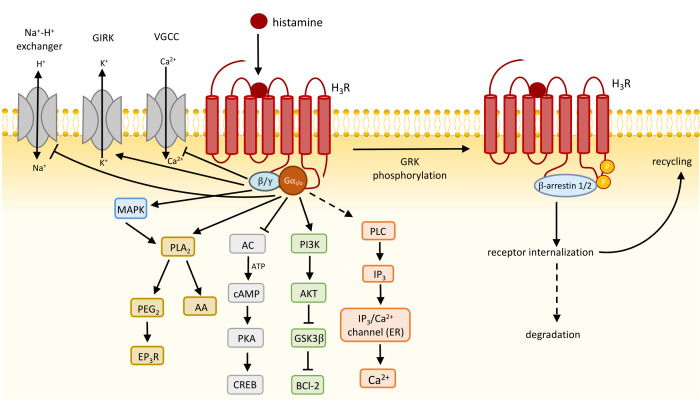
H_3_R-mediated intracellular signaling pathway and its regulation. H_3_R activation triggers signaling cascades via Gα_i/o_ or Gβγ subunits of heterotrimeric G_i/o_ protein to mediate intracellular response. Recruitment of β-arrestin1/2 to the GRK-phosphorylated H_3_R prevents further G protein coupling and directs the receptor towards internalization to prevent overstimulation.

The H_3_R-mediated activation of heterotrimeric Gα_i/o_ protein results in various intracellular responses: the inhibition of adenylyl cyclase (AC), voltage-gated calcium channels (VGCC), and the Na^+^/H^+^ exchanger; the activation of phosphatidylinositol 3-kinase (PI3K), mitogen-activated protein kinase (MAPK), phospholipase C (PLC), phospholipase A_2_ (PLA_2_), and G protein-gated inwardly rectifying potassium (GIRK) channels (vide infra) (Figure 3).

The H_3_R is one of the few GPCRs that shows constitutive activity in native tissues as revealed by the decrease in [^35^S]GTPγS accumulation to membranes from mouse cerebral cortex and various rat brain regions, including the cerebral cortex, striatum, hypothalamus, thalamus, hippocampus, and midbrain upon incubation with H_3_R inverse agonists FUB 465, ciproxifan, and thioperamide [37,71]. A similar decrease in H_3_R-mediated G protein activation was observed in hippocampus membranes from the ground squirrel upon incubation with the inverse agonist clobenpropit [72]. In addition, recombinant (over)expression of hH_3_R-445 in Chinese hamster ovary (CHO) cells resulted in a receptor level-dependent increase in constitutive [^35^S]GTPγS accumulation to activated G proteins, which can be inhibited by inverse agonists ciproxifan [71]. Similarly, increasing rH_3_R-445 or rH_3_R-413 density in CHO cells constitutively enhanced [^3^H]arachidonic acid release and reduced cAMP accumulation [37]. Moreover, agonists and inverse agonists induced an opposite bioluminescence resonance energy transfer (BRET) signal in an intramolecular BRET-based H_3_R biosensor in which the bioluminescent donor nanoluciferase (Nluc) was fused to the H_3_R C-terminal tail and ICL3 was substituted from Thr^229^ to Phe^348^ with the fluorescent acceptor HaloTag, indicating that the apo receptor is constitutively active and its conformation is shifted into an inactive versus more active one, respectively [73].

The short isoforms hH_3_R-373 and hH_3_R-365 display higher constitutive activity than hH_3_R-445 in transfected cells, resulting agonist-independent [^35^S]GTPγS binding to activated G proteins, inhibition of cAMP production, and the activation of extracellular signal-regulated kinases (ERK)1/2 MAPK, which could be reduced by inverse agonists [48,74,75]. Interestingly, agonists display higher potencies (pEC_50_) to activate these responses via hH_3_R-373 and hH_3_R-365 as compared to hH_3_R-445, whereas the opposite was observed for the inhibition of constitutive signaling by inverse agonists that have higher potencies on hH_4_R-445 [48,74]. These potency differences between agonists and inverse agonists for hH_3_R-365/373 versus hH_3_R-445 are in line with their binding affinities for these receptor isoforms [48,74,76] and follow the paradigm that agonists bind preferentially to (constitutive) active receptors, whereas inverse agonists display higher affinity for inactive receptor conformations [77]. In addition, the higher affinity of agonists for hH_3_R-365 as compared to hH_3_R-445 is associated with a slower deactivation rate of Gβγ-driven GIRK channel activity in a recombinant *Xenopus laevis* oocyte model following histamine washout, which was hypothesized to reflect slower ligand dissociation kinetics from a high versus lower affinity binding site and consequently leaving the ternary complex between agonist-bound receptor and G protein longer intact [78].

**Figure 4:**
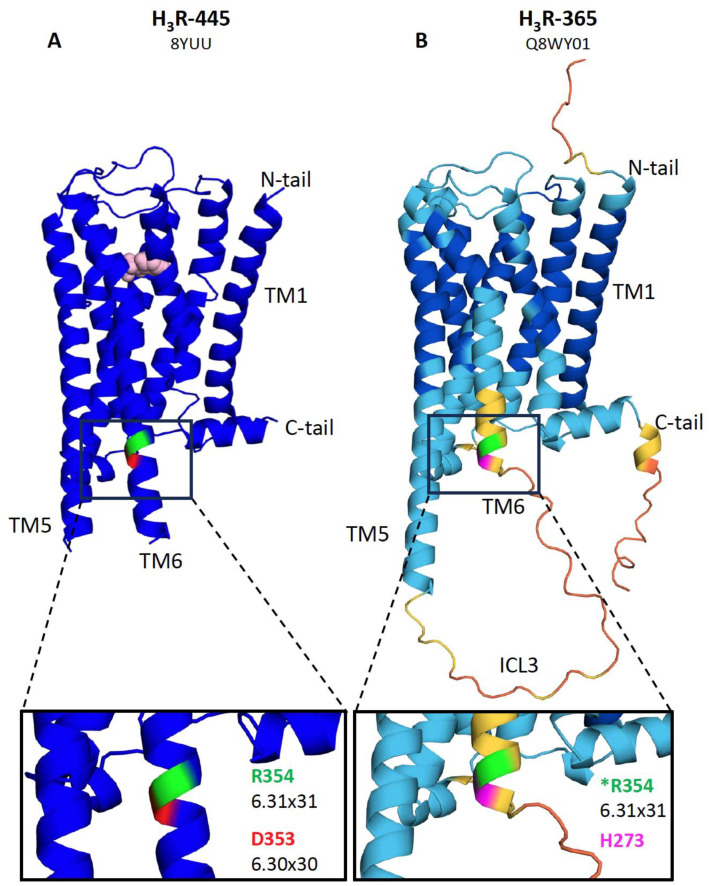
hH_3_R-365 has a shorter TM6 and ICL3 than hH_3_R-445. Comparison between cryo-EM structure of (**A**) hH_3_R-445 in complex with histamine (depicted as space filling molecule in light pink) (PDB: 8YUU) and predicted structures of (**B**) hH_3_R-365 (Q8WY01) by AlphaFold (https://alphafold.ebi.ac.uk; accessed on 5 April 2024) show the shorter ICL3 and TM6 in hH_3_R-365. The arginine at position 6.31 × 31 is present in both hH_3_R-445 (R^354^) and hH_3_R-365 (* indicated as R^354^ according to its number in the reference isoform 445) is depicted in green, whereas aspartate at position 6.30 × 30 in hH_3_R-445 is depicted in red. The 80-amino-acid deletion of segment DEF in H_3_R-365 places histidine 273 (indicated in magenta) at the end of segment C next to arginine at position 6.31 × 31. The AlphaFold confidence scores are indicated in blue (very high predicted local distance difference test (pLDDT) > 90), cyan (high 90 > pLDDT > 70), yellow (low > 70 > pLDDT > 50), and orange (very low pLDDT < 50) [82].

The hH_3_R-365/373 (AlphaFold accession code: Q8WY01) isoforms are the only ones in which, in addition to ICL3 (segments D and E), TM6 is shortened by approximately two helical turns (segment F) as compared to the cryo-EM structure of hH_3_R-445 (PDB accession code: 8YUU) and lacks the aspartate (D^353^ in hH_3_R-445) at position 6.30 × 30 (Figure 4) [79]. This aspartate (or glutamate) residue is conserved in many aminergic GPCRs and interacts with arginine at position 3.50 × 50 in the DRY motif within TM3 to form an ionic lock to maintain the receptor in an inactive conformation [80]. Disruption of this ionic lock by site-directed mutagenesis of the aspartate or glutamate at the 6.30 × 30 position increased constitutive activity in, for example, the β_2_-adrenergic receptor and H_1_R, and consequently increased agonist binding affinity [80,81]. Indeed, Ala-substitution of D^353^ in hH_3_R-445 resulted in slower GIRK deactivation rates than hH_3_R-445 upon histamine washout in oocytes, suggesting prolonged agonist binding, but not as slow as hH_3_R-365, indicating that the 80-amino-acid deletion has a larger effect on the receptor conformation [78]. Other isoforms with conserved 7TM, such as hH_3_R-415, hH_3_R-453, and hH_3_R-413, exhibit similar ligand affinity profiles to hH_3_R-445, which is in line with their comparable level of constitutive activity [74]. Interestingly, the shortest 7TM H_3_R isoform, hH_3_R-329a, exhibits increased binding affinities for agonists, while its binding affinity for inverse agonists is comparable to that of hH_3_R-445. The 116-amino-acid deletion (segments A–E) in hH_3_R-329a leaves only a four-amino-acid long ICL3 (i.e., ^343^SFTQ^346^ in the canonical hH_3_R-445 sequence), potentially hindering this receptor isoform from adopting a conformation that supports high-affinity binding of inverse agonists, despite the detection of inverse agonism [74].

### 4.2. Presynaptic H_3_R Inhibits Neurotransmitter Release and Synthesis

**Figure 5:**
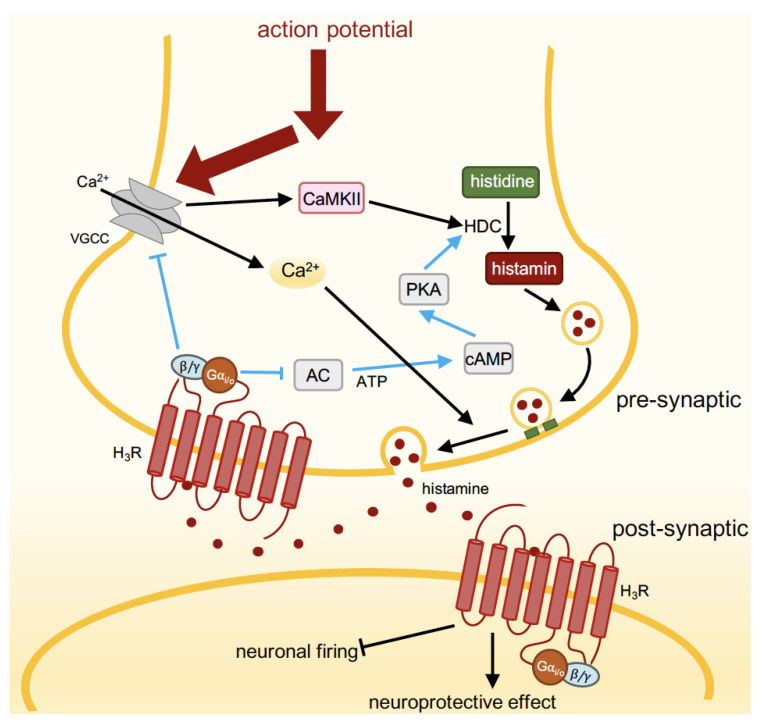
Presynaptic and postsynaptic H_3_R. Depolarization during an action potential opens voltage-gated calcium channels in the presynaptic terminal of an axon. The Ca^2+^ influx subsequently triggers calcium/calmodulin-dependent kinase II (CaMKII) to stimulate histamine synthesis by phosphorylating histidine decarboxylase (HDC) and triggering histamine release in the synaptic cleft. Histamine activates postsynaptic H_1_R, H_2_R and H_3_R, thereby modulating various neurological processes. Additionally, histamine activates presynaptic H_3_ autoreceptors to inhibit histamine synthesis and release by promoting the closure of VGCC and reducing the phosphorylation of HDC. These negative feedback loops are indicated in blue.

Neurons communicate with other cells by releasing neurotransmitters (e.g., histamine, dopamine, serotonin) in the synaptic cleft between these cells upon arrival of an axonal action potential and depolarization of the presynaptic terminal (Figure 5). This depolarization opens voltage-gated calcium channels (VGCC) resulting in an influx of Ca^2+^ ions. The increased intracellular Ca^2+^ concentrations subsequently drive the exocytotic machinery (involving SNARE proteins, SM proteins, and synaptotagim) to fuse vesicles that contain neurotransmitters with the presynaptic plasma membrane, releasing their content into the synaptic cleft [83,84]. In addition to the activation of their cognate receptors on postsynaptic cells, neurotransmitters can activate presynaptic autoreceptors to inhibit further neurotransmitter release by closing the VGCC in a Gβγ-dependent manner [85,86]. Indeed, stimulation of the H_3_R autoreceptor with agonists inhibited N- and P-type VGCC activity via pertussis toxin-sensitive heterotrimeric G_i/o_ proteins in depolarized rat tuberomammillary nucleus (TMN) histaminergic neurons [87], consequently attenuating histamine release from depolarized rat and mouse cortical synaptosomes [37]. Moreover, histamine release from these depolarized cortical synaptosomes is increased by H_3_R inverse agonists FUB 465 and thioperamide, confirming that native H_3_R is constitutively active and tonically inhibits histamine release [37]. Treatment of mice with the H_3_R inverse agonist ciproxifan (3 mg/kg) resulted in increased histamine release in the preoptic area and prefrontal cortex as detected with a genetically encoded fluorescent histamine (GRAB_HA_) sensor that was recombinantly expressed in these areas [88].

In non-histaminergic neurons, H_3_R acts as a presynaptic heteroreceptor and inhibits the release of other neurotransmitters such as acetylcholine, noradrenaline, serotonin, dopamine, glutamate, GABA, and neuropeptides via multiple G protein-mediated pathways (Table 2). For instance, H_3_R activation can result in a direct Gβγ-induced inhibition of VGCC to attenuate dopamine release, as detected in H_3_R-transfected nerve growth factor-differentiated rat pheochromacytoma cells, whose phenotype is close to that of sympathetic neurons [89]. The inhibition of norepinephrine exocytosis by H_3_R stimulation in guinea pig cardiac sympathetic nerve endings was found through the inhibition of the AC/protein kinase A (PKA) pathway to further phosphorylate VGCC [90]. The hyperpolarization of the cell membrane by GIRK channel activation, which allows potassium ion (K^+^) efflux from neurons, can potentially inhibit the subsequent opening of VGCC, which is necessary for further neurotransmitter release [91]. It has been shown that the GIRK channel blocker tertiapin-Q has effectively prevented the H_3_R agonist immepip-induced inhibition of glutamate release in a rat corticostriatal synapse as measured by decreased paired-pulse ratios [92]. Furthermore, a mechanistic connection between H_3_R-induced MAPK activation and subsequent PLA_2_ phosphorylation, which initiates anti-exocytotic processes, such as prostaglandin E2 (PGE_2_) production to activate prostaglandin EP_3_ receptor (EP_3_R) and block VGCC, ultimately reduces norepinephrine release in guinea pig heart synaptosomes [93]. However, whether the MAPK-PLA_2_-PGE_2_-EP_3_R axis and GIRK channels contribute to autoreceptor H_3_R-mediated histamine release remains unclear.

In addition, the activation of the presynaptic H_3_R autoreceptor suppresses the activity of histidine decarboxylase (HDC) and consequently histamine synthesis in the rat cerebral cortex via the inhibition of calcium/calmodulin-dependent protein kinase type II (CaMKII) and PKA-mediated phosphorylation by the Gβγ- and Gα_i/o_-mediated inhibition of N- and P-type VGCC and adenylyl cyclase, respectively [112,113]. Importantly, H_3_R inverse agonists such as thioperamide and clobenpropit enhanced histamine synthesis, indicating that the constitutive activity of H_3_R contributes to the negative feedback regulation of histamine synthesis [112,113]. Notably, H_3_R activation can also inhibit dopamine synthesis in the rat nucleus accumbens by the inhibition of the PKA pathway to attenuate tyrosine hydroxylase phosphorylation [65], thereby reducing the available amounts of dopamine for release.

Interestingly, the autoreceptor function of rat H_3_R is proposed to be carried out by the short isoform. The stereoselectivity of N^α^Me-αClMeHA and R(-)sopromidine enantiomers on cAMP formation on CHO cells transfected with rH_3_R-413 was found to be more similar to the effects on the rat cortex, striatum, and hypothalamus, which are areas rich in autoreceptors [114]. Other GPCRs, such as the short dopamine D_2_ receptor (D_2_R), with a 29-amino-acid deletion in the third intracellular loop, also function as an autoreceptor.

### 4.3. Postsynaptic H_3_R Function and Downstream Effects

Activation of postsynaptic H_3_R was found to diminish the firing rate of melanin-concentrating hormone-producing neurons [115]. In substantia nigra pars reticulata (SNr) GABA projection neurons, H_3_R activation hyperpolarized and suppressed firing frequency, consequently decreasing the intensity of basal ganglia output [116]. The reduced firing frequency by H_3_R is related to the reduced phosphorylation level of ERK and the increased A-type K^+^ current [117,118]. A more recent study, using a chimeric H_3_R protein in which ICL3 is fused with extracellular and transmembrane domains of rhodopsin to convey light responsiveness, demonstrated that postsynaptic H_3_R activation in ventral basal forebrain cholinergic neurons is responsible for the inhibition of contextual fear memory retrieval via suppressing the firing frequency [119].

Downstream effects mediated by H_3_R contribute to neurological processes such as promoting neurogenesis or exerting neuroprotective effects. For instance, thioperamide can protect primary neurons against oxygen–glucose deprivation-induced injury and promote the proliferation of the neural stem cell line NE-4C through stimulation of downstream cAMP response element binding protein (CREB) phosphorylation, aligning with the constitutive activity of H_3_R in native tissues [120]. H_3_R activation by the agonist imetit improves the viability of mouse primary cortical neurons that are impaired by oxygen–glucose deprivation/reoxygenation conditions via promoting ERK1/2 phosphorylation in a PTX-sensitive manner [121]. Besides, H_3_R activation protects cultured rat and mouse cortical neurons from neurotoxic insults by increasing the expression of the anti-apoptotic protein BCl-2 via the Akt–glycogen synthase kinase (GSK) 3β axis [122], which is constitutively activated via G_i/o_ proteins and phosphoinositide-3-kinase (PI3K) in SK-N-MC cells recombinantly expressing hH_3_R-445 [123].

### 4.4. H_3_R Dimerization

In the striatum, H_3_R is present on the afferent terminals of glutamatergic and dopaminergic neurons and acts as presynaptic heteroreceptor (vide supra) to negatively regulate glutamate and dopamine release [64,124,125]. However, the majority of striatal H_3_Rs are expressed as postsynaptic receptors on efferent striato-nigral and striato-pallidal GABAergic medium spiny neurons (MSN) [64,126], where they co-localize in close proximity with D_1_R and D_2_R, respectively, as shown by an antibody-based proximity labeling assay (PLA) in rodent striata [127,128,129]. Co-immunoprecipitation of H_3_R with either D_1_R or D_2_R using specific antibodies from rodent striatal lysates provided additional evidence that these receptors might physically interact in MSNs [126,127,128,129]. Further support that H_3_R might specifically interact with D_1_R and D_2_R has been provided by saturable bioluminescence resonance energy transfer (BRET) between one receptor fused to luciferase (BRET donor) expressed at a fixed level in combination with increasing levels of the other receptor fused to fluorescent protein (BRET acceptor) in heterologous HEK293(T) cell lines [130,131].

GPCRs can modulate each other’s trafficking, ligand binding, and/or signaling properties when forming dimeric or multimeric complexes [132,133]. Negative binding cooperativity was observed within H_3_R/D_1_R and H_3_R/D_2_R heteromers, with H_3_R agonists decreasing the binding affinity of D_1_R and D_2_R agonists [130,131], which might contribute to the H_3_R agonist-mediated attenuation of D_1_R and D_2_R agonist-induced locomotor activity in mice [127,130]. In contrast, H_3_R inverse agonists potentiated D_1_R and D_2_R-mediated locomotor activity in response to their agonists, SKF38393 and quinpirole, respectively, in mice with a depletion in endogenous striatal dopamine, indicating that H_3_R constitutively attenuates the responsiveness of the associated D_1_R or D_2_R protomers [130]. Stimulation of H_3_R attenuated D_2_R-mediated Akt–GSK3β signaling in striato-pallidal MSNs in response to D_2_R agonists in a β-arrestin2-dependent manner [127]. Interestingly, D_1_R agonists increased cAMP levels in cell lines expressing only the G_s_-coupled D_1_R but decreased forskolin-induced cAMP levels in cell lines co-expressing D_1_R and the G_i/o_-coupled H_3_R, suggesting that the D_1_R/H_3_R heteromer signals through the H_3_R protomer [128,131]. Cells recombinantly expressing D_1_R showed agonist-induced extracellular signal-regulated kinase 1/2 (ERK1/2) phosphorylation, whereas cells that only expressed H_3_R did not activate this response [131]. However, cells co-expressing D_1_R and H_3_R showed pERK1/2 in response to stimulation with D_1_R (SKF 38393 or SKF 81297) or H_3_R (RAMH or imetit) agonists, which could be cross-antagonized by selective D_1_R (SCH23390) and H_3_R (thioperamide) antagonists [128,131]. Similarly, postsynaptic D_1_R/H_3_R-mediated signaling to ERK1/2 and cross-antagonism was observed in striatal slices obtained from rats and wild-type mice but not in striata from transgenic mice that lack D_1_R. Importantly, the disruption of D_1_R/H_3_R heteromers in immortalized striatal cells using a synthetic transmembrane 5 peptide (TAT-TM5) abolished cross-antagonism of the D_1_R agonist (SKF 81297)-induced pERK1/2 by the H_3_R antagonist thioperamide [129]. In addition, striatal cell death involving the p38 apoptotic pathway, upon overactivation by the D_1_R agonist SKF 81297 (>30 μM), can be cross-antagonized by the H_3_R antagonist thioperamide, which was abolished upon disruption of the D_1_R/H_3_R heteromer by TAT-TM5 or the downregulation of H_3_R using shRNA [129].

In a preclinical Huntington’s disease (HD) mice model (Hdh^Q7^/^Q111^ knock-in), D_1_R-H_3_R heteromers were detected by PLA in the striatum, cerebral cortex, and hippocampus slices at 2–4 months of age but were undetectable at early disease states at 6–8 months of age [129]. Indeed, thioperamide can prevent D_1_R agonist SKF 81297-induced apoptosis in striatal, cortical, and hippocampal organotypic cultures from HD mice at four but not eight months of age, confirming that D_1_R/H_3_R heteromerization is required for this cross-antagonism [129]. In humans, D_1_R/H_3_R heteromers are present in striatal (caudate putamen) slices of control individuals and individuals with low-grade (0, 1, and 2) HD, but are almost absent in individuals with high-grade (3 and 4) HD [129]. In immortalized mouse striatal cells, the D_1_R agonist SKF 81297 reduces D_1_R/H_3_R heteromerization, which could be prevented by pretreatment with thioperamide [129]. Moreover, the chronic treatment of HD mice with thioperamide has been shown to prevent the loss of D_1_R/H_3_R heteromers and cognitive and motor learning deficits at early disease states, but not when D_1_R/H_3_R heteromers were already lost in late disease states [129].

Interestingly, cocaine can disrupt the cross-antagonism of the D_1_R agonist SKF 38393-induced pERK1/2 and apoptosis in the rodent striatum by thioperamide in a sigma-1 receptor (σ_1_R)-dependent manner, which can be blocked by pretreatment with σ_1_R antagonist PD 144418 [128]. BRET and sequential resonance energy experiments in transfected cells revealed that σ_1_R interacts with the D_1_R protomer in the D_1_R/H_3_R heteromer, whereas their close proximity was confirmed in the rodent striatum by PLA and the co-immunoprecipitation of σ_1_R and H_3_R with D_1_R [129]. Hence, antagonizing σ_1_R restores the protective effect of H_3_R on D_1_R signaling in cocaine-induced cell death.

Postsynaptic D_1_R/H_3_R heteromers have also been detected in the rodent cerebral cortex, where they interact with ionotropic N-methyl-D-aspartate (NMDA) glutamate receptors [134]. Importantly, H_3_R antagonist thioperamide cross-antagonized NMDA- and D_1_R agonist-induced excitotoxic cell death in rodent cortical cultures. In addition, both the D_1_R and H_3_R antagonist prevented neurodegeneration resulting from Aβ peptide toxicity in the context of Alzheimer’s disease [134].

Presynaptic H_3_R physically interacts with adenosine 2A receptors (A_2A_R) in the terminals of striatopallidal MSNs, as revealed by co-immunoprecipitation experiments [135]. H_3_R decreased the binding affinity of the A_2A_R agonist in globus pallidus synaptosomal membranes by two-fold, whereas a two-fold increase in affinity was observed in the opposite direction, suggesting allosteric interaction within the H_3_R/A_2A_R heteromer [136]. The G_s_-coupled A_2A_R enhances the VGCC/Ca^2+^-dependent GABA release from depolarized striatopallidal synaptosomes via the cAMP/PKA pathway, which is counteracted by H_3_R via the Ga_i/o_-mediated inhibition of adenylyl cyclase [136].

H_3_R homodimerization is shown in transfected HEK293 cells and cortical neurons using BRET and co-immunoprecipitation, whereas Western blot analysis of rat cortices, cerebella, and hypothalamus membranes suggests the presence of both monomers and dimers [137]. Interestingly, H_3_R agonists and inverse agonists induced a concentration-dependent decrease and increase in BRET amplitude, which was more pronounced in the cortical neurons as compared to HEK293 cells [137]. These BRET changes were interpreted as conformational changes within dimers and not the association or dissociation of dimers, which was supported by the fact that agonists did not induce dimer dissociation in Western blot analysis.

The detection of endogenous H_3_R heteromers in tissue slices by Co-IP and PLA methods does not discriminate between H_3_R isoforms. Hitherto, the dimerization of other H_3_R isoforms than the canonical H_3_R-445 in recombinant cells has not been reported to our best knowledge, whereas the ex vivo studies suggest potential interactions. Three rat H_3_R isoforms (rH_3_R-497, rH_3_R-465, and rH_3_R-449) that have a unique amino acid sequence after TM6, and consequently lack the conserved TM7 and C-tail, are unable to bind H_3_R ligands or inhibit adenylyl cyclase activity [41]. However, these three isoforms have a dominant negative effect on the trafficking of rH_3_R-445 and reduces its expression at the cell surface, presumably by engaging into dimers that are retained intracellularly, as observed for truncated histamine H_4_ receptors (H_4_R) and α_1A_-adrenergic receptor splice isoforms [53,138].

### 4.5. Regulation of H_3_R Signaling

Agonist-bound GPCRs are phosphorylated at their C-terminal tail and/or intracellular loops by GPCR kinases (GRKs), resulting in the recruitment of cytosolic β-arrestin1 and/or β-arrestin2 [139], which desensitizes the receptor by hindering further G protein coupling and preventing continuous responsiveness [140]. In addition, β-arrestins act as a scaffold for several proteins involved in the endocytic process, including clathrin, to facilitate receptor internalization.

Some ex vivo evidence indicates that pre-exposure to H_3_R agonists may induce the desensitization of subsequent responsiveness to a secondary stimulation with an agonist. For instance, in the guinea pig ileum, where H_3_R activation attenuates electrically-induced contraction primarily through the inhibition of acetylcholine release from postganglionic cholinergic neurons, prior exposure to the agonist RAMH has led to reduced potency and efficacy in subsequent agonist applications [141]. Besides, the specific binding of [^3^H]NAMH to membranes from rat striatal slides was observed to decrease after pre-treatment of the agonist immepip, suggesting that H_3_R is internalized/downregulated [142]. Furthermore, the reduction in functional responses, including cAMP signal and [^35^S]GTPγS accumulation, as well as [^3^H]NAMH binding, was prevented by culturing CHO cells transfected with H_3_R in a hypertonic medium or incubation at 4 °C, which is known to affect clathrin-dependent endocytosis [143], indicating the involvement of clathrin-dependent internalization in H_3_R desensitization. GRK2 is suggested to play a major role in this process, as downregulation of GRK2 expression by small interfering RNA (siRNA) in CHO cells attenuates the desensitization of the cAMP signal and the reduction of [^3^H]NAMH binding due to prolonged agonist exposure [143].

Notably, differential desensitization dynamics are observed between H_3_R isoforms hH_3_R-365 or hH_3_R-445. Studies in CHO cells transfected with either hH_3_R-365 or hH_3_R-445 have revealed that hH_3_R-365 desensitizes more rapidly compared to hH_3_R-445. However, despite its quicker onset, hH_3_R-365 reaches a lower maximum extent of desensitization and resensitization. This suggests that while hH_3_R-365 can quickly become unresponsive to stimuli, it also recovers its responsiveness more swiftly than hH_3_R-445 [144]. Furthermore, hH_3_R-415 and hH_3_R-445 have been shown to effectively recruit β-arrestin2 upon agonist stimulation, facilitating their desensitization and internalization processes. In contrast, hH_3_R-365 and hH_3_R-329a demonstrate higher efficacy and potency in recruiting β-arrestin2. This indicates that these isoforms might have a stronger or more immediate regulatory response to agonist binding, leading to more pronounced desensitization [75].

In addition, H_3_R-445 responsiveness to agonist stimulation is desensitized in CHO-cells by protein kinase C-mediated phosphorylation following the stimulation of G_q_-coupled endogenous purinergic P2Y2 receptors with adenosine 5’-triphosphate (ATP), indicating potential cross-regulation between GPCRs [145]. However, hitherto, no heterologous desensitization of H_3_R has been reported in native tissue.

### 4.6. Isoform Signaling Bias

Alternative splicing of the intracellular ICL3 and/or C-terminal tail can alter the conformational activity state of the receptor and consequently indirectly affect ligand binding to the extracellular side of the 7TM domain, with, for example, a 20- and 32-fold lower binding affinity of pitolisant on hH_3_R-373 and hH_3_R-365, respectively, as compared to the less constitutively active hH_3_R-445 (vide supra). This significant difference in binding affinity implies the necessity of a more detailed analysis of the signaling capacities of H_3_R isoforms for future H_3_R drug discovery efforts.

Isoform-specific functional properties extend beyond binding selectivity. For example, the H_3_R agonists impentamine and dimethyl-impentamine act as partial agonists on H_3_R-453, H_3_R-445, H_3_R-415, H_3_R-413, H_3_R-373, and H_3_R-329a, but are full agonists on H_3_R-365, with the same intrinsic activity as histamine, with comparable binding affinities for hH_3_R-445 and hH_3_R-365 [74]. Interestingly, hH_3_R-365 was also found to be unable to further activate GSK3β phosphorylation in CHO-K1 transfected cells with agonist stimulation [146]. Additionally, certain isoforms are specifically modulated in a ligand-directed manner. For instance, the H_3_R agonists proxyfan and iodoproxyfan elicited a robust response in increasing intracellular Ca^2+^ concentrations but failed to elicit a response for hH_3_R-365, indicating the potential for “isoform-biased” agonists [146]. The selectivity of G protein coupling by GPCR is an intricate process that is heavily influenced by the structural variations within the receptor, particularly in the intracellular loops and transmembrane helices. ICL3, which connects TM5 and TM6, plays a pivotal role in this selectivity as there is a dynamic conformational equilibrium of ICL3 between blocking and exposing the G protein-binding site that allows for the autoregulation of receptor activity [47]. Shorter isoforms with a truncated ICL3 might favor coupling with different subtypes of G proteins compared to longer isoforms, which have a more extended ICL3 that can stabilize different conformations. This can result in variations in signaling efficiency and receptor autoinhibition, as seen in other GPCRs, like the dopamine D_2_ receptor, where the long isoform (D_2_L) has a higher efficacy of canonical signaling compared to the short isoform (D_2_S) with a 29-amino-acid deletion in ICL3 [46,147]. Given the varying lengths of ICL3 in H_3_R isoforms, these isoforms might exhibit distinct G protein coupling profiles, which presumably leads to differential downstream functional outcomes in addition to their G_i/o_-mediated inhibition of adenylyl cyclase activity [74]. Furthermore, structural analyses from cryo-EM studies of H_3_R (PDB ID: 8YUU and 8YUV) show that the lengths of TM5 and TM6 are longer than in H_1_R and H_2_R. Machine learning analysis of 98 homology models from GPCRdb revealed that GPCRs with a long TM5 length are more likely to couple to G_i/o_ proteins as compared to those with a shorter or tilted TM5. Interestingly, TM5 of hH_3_R-329a is 1.5 helical turns shorter as compared to the hH_3_R-445 cryo-EM structure. Agonist stimulation of hH_3_R-329a did not activate pAKT T^308^/S^473^ in CHO-K1-transfected cells, whereas a robust response was observed in hH_3_R-415, hH_3_R-365, and hH_3_R-445 [75]. However, whether this is related to its G protein selectively remains to be investigated. Exchange of the intracellular half of TM5, TM6, and ICL3 (P^210^ to P^373^) of H_3_R with the corresponding section of the G_s_-coupled H_2_R (P^194^ to P^249^) shifted its coupling from G_i_ to G_s_, confirming the role of these domains in G protein selectivity [79]. The switch from G_i/o_ to G_s_ signaling might result in changes downstream for signaling cascades, potentially leading to varied therapeutic effects and side effects, as observed in the μ-opioid receptor (MOR), for instance. The MOR-1D isoform with an extended C-terminus, in comparison to the canonical isoform MOR-1, has been implicated in morphine-induced itch as a side effect [148]. Additionally, chronic exposure to morphine results in the upregulation of MOR isoforms MOR-1B2 and MOR-1C1. These variants undergo phosphorylation at C-terminal sites that are not present in the canonical MOR-1 isoform, which is associated with a shift from the predominantly inhibitory G_i/o_ coupling pathway to the stimulatory G_s_ pathway. This shift leads to changes in cellular responses, and this switch can contribute to the development of tolerance, dependence, and other side effects, such as opioid-induced hyperalgesia or itch [149].

The expression of truncated 6TM GPCR isoforms has been extensively documented in human and rodents, including H_3_R and the delta-opioid receptor [150]. These 6TM isoforms exert their function via interacting with intracellular compartments or affecting other 7TM isoforms. Studying H_3_R isoforms might uncover new intracellular signaling or regulation mechanisms, such as dimerization that may contribute to overall receptor function. Understanding the structural determinants of G protein selectivity in H_3_R isoforms is crucial for developing targeted therapies that can modulate specific receptor activities in pathological states.

## 5. Discussion

In this review, we have discussed the existence of the H_3_R isoforms that have been identified so far within and across species, which expands the complexity of H_3_R function in vivo. The research of H_3_R isoforms holds significant promise and is crucial for advancing our understanding of H_3_R functionality and its implications for drug development. However, the identification of various H_3_R isoforms across different species and tissues indicates complexity in H_3_R-mediated signaling that remains underexplored. As anticipated, the canonical H_3_R-445 isoform is predominantly expressed in the brain. However, one compelling reason to study H_3_R isoforms is their potential differential expression in pathological states, as shown by RNA sequencing. Currently, there is limited research on how and where these isoform proteins are expressed in diseased tissues, which could provide insights into their roles in disease progression and response to treatment. Understanding these patterns could lead to the development of isoform-specific drugs, which might offer more precise therapeutic interventions with potentially fewer side effects. In addition, investigation of the physiological roles of individual isoforms in more relevant biological in vivo and/or ex vivo contexts might provide clearer understanding of their functional significance. In particular, considering the observed differences in the constitutive signaling and ligand affinities between some of the isoforms. This knowledge will enhance our ability to design drugs that can specifically target desired signaling pathways, thereby improving therapeutic outcomes. Hence, the development of selective tool ligands and or antibodies to unambiguously identify which isoform protein is mediating an observed effect in native tissue has so far been challenging but is key for our in-depth understanding of their function and therapeutic potential.

## Figures and Tables

**Table 2:** Presynaptic H_3_R-mediated neurotransmitter release in various species.

Transmitter	Species	Tissue	Pathway	References
Histamine	Human	Neocortex	↓AC, ↓cAMP	[94]
	Mouse	Neocortex		[37]
	Rat	Neocortex		[37,94,95]
	Guinea pig	Cardiac synaptosomes		[96]
	Hamster	Hypothalamus		[34]
	Zebrafish	Hypothalamus		[97]
Acetylcholine	Rat	Cortex	↓VGCC	[50,98]
	Guinea pig	Ileum		[99]
Noradrenaline	Human	Neocortex		[100]
	Mouse	Neocortex		[101,102]
	Rat	Neocortex		[103,104]
	Guinea pig	Cardiac synaptosomes	↓AC/↓PKA/↓VGCC; ↑MAPK/↑PLA_2_/↑PEG_2_/↑EP_3_R/↓VGCC	[90,93]
Serotonin	Guinea pig	Mesenteric artery	↓PKA/↓VGCC	[105]
	Rat	Neocortex	↓AC, ↓cAMP	[104,106,107]
Dopamine	Mouse	Striatum	↓AC, ↓cAMP	[108]
Glutamate	Rat	Striatum	↑GIRK, ↓VGCC	[92]
GABA	Rat	Striatum	↓AC, ↓cAMP ↓PKA/↓VGCC	[109]
Neuropeptides	Rat	Dura mater	↓AC, ↓cAMP ↓PKA/↓VGCC	[110,111]
	Guinea pig	Dura mater		[110]

AC; adenylate cyclase, cAMP; cyclic adenosine monophosphate, PKA; protein kinase A, VGCC; voltage-gated calcium channels; GIRK; G protein-gated inwardly rectifying potassium.

## Data Availability

Splice-O-Mat (https://tools.hornlab.org/Splice-O-Mat/ accessed on 23 April 2024), Adult GTEx (https://gtexportal.org/home/transcriptPage accessed on 23 April 2024), Human Protein Atlas (https://www.proteinatlas.org/about/download accessed on 23 April 2024), AlphaFold (https://alphafold.ebi.ac.uk accessed on 5 April 2024).

## References

[1] Marasco L.E., Kornblihtt A.R. (2023). The physiology of alternative splicing. Nat. Rev. Mol. Cell Biol..

[2] Olivieri J.E., Dehghannasiri R., Wang P.L., Jang S., de Morree A., Tan S.Y., Ming J., Ruohao Wu A., Tabula Sapiens C., Quake S.R. (2021). RNA splicing programs define tissue compartments and cell types at single-cell resolution. eLife.

[3] Markovic D., Challiss R.A. (2009). Alternative splicing of G protein-coupled receptors: Physiology and pathophysiology. Cell. Mol. Life Sci..

[4] Hauser A.S., Attwood M.M., Rask-Andersen M., Schioth H.B., Gloriam D.E. (2017). Trends in GPCR drug discovery: New agents, targets and indications. Nat. Rev. Drug Discov..

[5] Mele M., Ferreira P.G., Reverter F., DeLuca D.S., Monlong J., Sammeth M., Young T.R., Goldmann J.M., Pervouchine D.D., Sullivan T.J. (2015). Human genomics. The human transcriptome across tissues and individuals. Science.

[6] Marti-Solano M., Crilly S.E., Malinverni D., Munk C., Harris M., Pearce A., Quon T., Mackenzie A.E., Wang X., Peng J. (2020). Combinatorial expression of GPCR isoforms affects signalling and drug responses. Nature.

[7] Panula P., Chazot P.L., Cowart M., Gutzmer R., Leurs R., Liu W.L., Stark H., Thurmond R.L., Haas H.L. (2015). International Union of Basic and Clinical Pharmacology. XCVIII. Histamine Receptors. Pharmacol. Rev..

[8] Cheng L., Liu J., Chen Z. (2021). The Histaminergic System in Neuropsychiatric Disorders. Biomolecules.

[9] Harwell V., Fasinu P.S. (2020). Pitolisant and Other Histamine-3 Receptor Antagonists-An Update on Therapeutic Potentials and Clinical Prospects. Medicines.

[10] Syed Y.Y. (2016). Pitolisant: First Global Approval. Drugs.

[11] Ligneau X., Perrin D., Landais L., Camelin J.C., Calmels T.P., Berrebi-Bertrand I., Lecomte J.M., Parmentier R., Anaclet C., Lin J.S. (2007). BF2.649 [1-3-[3-(4-Chlorophenyl)propoxy]propylpiperidine, hydrochloride], a nonimidazole inverse agonist/antagonist at the human histamine H3 receptor: Preclinical pharmacology. J. Pharmacol. Exp. Ther..

[12] Urquhart L. (2019). FDA new drug approvals in Q3 2019. Nat. Rev. Drug Discov..

[13] Coge F., Guenin S.P., Audinot V., Renouard-Try A., Beauverger P., Macia C., Ouvry C., Nagel N., Rique H., Boutin J.A. (2001). Genomic organization and characterization of splice variants of the human histamine H3 receptor. Biochem. J..

[14] Gallagher M., Yates S.L. (2003). Histamine H3 Receptor Polynucleotides. WO Patent.

[15] Lovenberg T.W., Roland B.L., Wilson S.J., Jiang X., Pyati J., Huvar A., Jackson M.R., Erlander M.G. (1999). Cloning and functional expression of the human histamine H3 receptor. Mol. Pharmacol..

[16] Nakamura T., Itadani H., Hidaka Y., Ohta M., Tanaka K. (2000). Molecular cloning and characterization of a new human histamine receptor, HH4R. Biochem. Biophys. Res. Commun..

[17] Tardivel-Lacombe J., Morisset S., Gbahou F., Schwartz J.C., Arrang J.M. (2001). Chromosomal mapping and organization of the human histamine H3 receptor gene. Neuroreport.

[18] Tsui P. (2001). Human Histamine H3 Gene Variant-2. WO Patent.

[19] Tsui P. (2001). Human Histamine H3 Gene Variant-3. WO Patent.

[20] Wellendorph P., Goodman M.W., Burstein E.S., Nash N.R., Brann M.R., Weiner D.M. (2002). Molecular cloning and pharmacology of functionally distinct isoforms of the human histamine H(3) receptor. Neuropharmacology.

[21] GTEx-Consortium (2020). The GTEx Consortium atlas of genetic regulatory effects across human tissues. Science.

[22] Kuhn C.K., Stenzel U., Berndt S., Liebscher I., Schoneberg T., Horn S. (2024). The repertoire and structure of adhesion GPCR transcript variants assembled from publicly available deep-sequenced human samples. Nucleic Acids Res..

[23] Thakkar M.M. (2011). Histamine in the regulation of wakefulness. Sleep Med. Rev..

[24] Lin W., Xu L., Zheng Y., An S., Zhao M., Hu W., Li M., Dong H., Li A., Li Y. (2023). Whole-brain mapping of histaminergic projections in mouse brain. Proc. Natl. Acad. Sci. USA.

[25] Panula P., Nuutinen S. (2013). The histaminergic network in the brain: Basic organization and role in disease. Nat. Rev. Neurosci..

[26] Khouma A., Moeini M.M., Plamondon J., Richard D., Caron A., Michael N.J. (2023). Histaminergic regulation of food intake. Front. Endocrinol..

[27] Connelly W.M., Shenton F.C., Lethbridge N., Leurs R., Waldvogel H.J., Faull R.L., Lees G., Chazot P.L. (2009). The histamine H4 receptor is functionally expressed on neurons in the mammalian CNS. Br. J. Pharmacol..

[28] Fang Q., Xicoy H., Shen J., Luchetti S., Dai D., Zhou P., Qi X.R., Martens G.J.M., Huitinga I., Swaab D.F. (2021). Histamine-4 receptor antagonist ameliorates Parkinson-like pathology in the striatum. Brain Behav. Immun..

[29] Shan L., Martens G.J.M., Swaab D.F. (2022). Histamine-4 Receptor: Emerging Target for the Treatment of Neurological Diseases. Curr. Top. Behav. Neurosci..

[30] Sanna M.D., Ghelardini C., Thurmond R.L., Masini E., Galeotti N. (2017). Behavioural phenotype of histamine H(4) receptor knockout mice: Focus on central neuronal functions. Neuropharmacology.

[31] Arrang J.M., Garbarg M., Schwartz J.C. (1983). Auto-inhibition of brain histamine release mediated by a novel class (H3) of histamine receptor. Nature.

[32] Chen J., Liu C., Lovenberg T.W. (2003). Molecular and pharmacological characterization of the mouse histamine H3 receptor. Eur. J. Pharmacol..

[33] Lovenberg T.W., Pyati J., Chang H., Wilson S.J., Erlander M.G. (2000). Cloning of rat histamine H(3) receptor reveals distinct species pharmacological profiles. J. Pharmacol. Exp. Ther..

[34] Barrett P., Ross A.W., Balik A., Littlewood P.A., Mercer J.G., Moar K.M., Sallmen T., Kaslin J., Panula P., Schuhler S. (2005). Photoperiodic regulation of histamine H3 receptor and VGF messenger ribonucleic acid in the arcuate nucleus of the Siberian hamster. Endocrinology.

[35] Strakhova M.I., Fox G.B., Carr T.L., Witte D.G., Vortherms T.A., Manelli A.M., Miller T.R., Yao B.B., Brioni J.D., Esbenshade T.A. (2008). Cloning and characterization of the monkey histamine H3 receptor isoforms. Eur. J. Pharmacol..

[36] Yao B.B., Sharma R., Cassar S., Esbenshade T.A., Hancock A.A. (2003). Cloning and pharmacological characterization of the monkey histamine H3 receptor. Eur. J. Pharmacol..

[37] Morisset S., Rouleau A., Ligneau X., Gbahou F., Tardivel-Lacombe J., Stark H., Schunack W., Ganellin C.R., Schwartz J.C., Arrang J.M. (2000). High constitutive activity of native H3 receptors regulates histamine neurons in brain. Nature.

[38] Morisset S., Sasse A., Gbahou F., Heron A., Ligneau X., Tardivel-Lacombe J., Schwartz J.C., Arrang J.M. (2001). The rat H3 receptor: Gene organization and multiple isoforms. Biochem. Biophys. Res. Commun..

[39] Tardivel-Lacombe J., Rouleau A., Heron A., Morisset S., Pillot C., Cochois V., Schwartz J.C., Arrang J.M. (2000). Cloning and cerebral expression of the guinea pig histamine H3 receptor: Evidence for two isoforms. Neuroreport.

[40] Rouleau A., Heron A., Cochois V., Pillot C., Schwartz J.C., Arrang J.M. (2004). Cloning and expression of the mouse histamine H3 receptor: Evidence for multiple isoforms. J. Neurochem..

[41] Bakker R.A., Lozada A.F., van Marle A., Shenton F.C., Drutel G., Karlstedt K., Hoffmann M., Lintunen M., Yamamoto Y., van Rijn R.M. (2006). Discovery of naturally occurring splice variants of the rat histamine H3 receptor that act as dominant-negative isoforms. Mol. Pharmacol..

[42] Drutel G., Peitsaro N., Karlstedt K., Wieland K., Smit M.J., Timmerman H., Panula P., Leurs R. (2001). Identification of rat H3 receptor isoforms with different brain expression and signaling properties. Mol. Pharmacol..

[43] Peitsaro N., Sundvik M., Anichtchik O.V., Kaslin J., Panula P. (2007). Identification of zebrafish histamine H1, H2 and H3 receptors and effects of histaminergic ligands on behavior. Biochem. Pharmacol..

[44] Sjostedt E., Zhong W., Fagerberg L., Karlsson M., Mitsios N., Adori C., Oksvold P., Edfors F., Limiszewska A., Hikmet F. (2020). An atlas of the protein-coding genes in the human, pig, and mouse brain. Science.

[45] Medhurst A.D., Atkins A.R., Beresford I.J., Brackenborough K., Briggs M.A., Calver A.R., Cilia J., Cluderay J.E., Crook B., Davis J.B. (2007). GSK189254, a novel H3 receptor antagonist that binds to histamine H3 receptors in Alzheimer’s disease brain and improves cognitive performance in preclinical models. J. Pharmacol. Exp. Ther..

[46] Reiner-Link D., Madsen J.S., Gloriam D.E., Brauner-Osborne H., Hauser A.S. (2024). Differential G protein activation by the long and short isoforms of the dopamine D(2) receptor. Br. J. Pharmacol..

[47] Sadler F., Ma N., Ritt M., Sharma Y., Vaidehi N., Sivaramakrishnan S. (2023). Autoregulation of GPCR signalling through the third intracellular loop. Nature.

[48] Bongers G., Krueger K.M., Miller T.R., Baranowski J.L., Estvander B.R., Witte D.G., Strakhova M.I., van Meer P., Bakker R.A., Cowart M.D. (2007). An 80-amino acid deletion in the third intracellular loop of a naturally occurring human histamine H3 isoform confers pharmacological differences and constitutive activity. J. Pharmacol. Exp. Ther..

[49] Esbenshade T.A., Strakhova M.I., Carr T.L., Sharma R., Witte D.G., Yao B.B., Miller T.R., Hancock A.A. (2006). Differential CNS expression and functional activity of multiple human H(3) receptor isoforms. Inflamm. Res..

[50] Arrang J.M., Drutel G., Schwartz J.C. (1995). Characterization of histamine H3 receptors regulating acetylcholine release in rat entorhinal cortex. Br. J. Pharmacol..

[51] Strausberg R.L., Feingold E.A., Grouse L.H., Derge J.G., Klausner R.D., Collins F.S., Wagner L., Shenmen C.M., Schuler G.D., Altschul S.F. (2002). Generation and initial analysis of more than 15,000 full-length human and mouse cDNA sequences. Proc. Natl. Acad. Sci. USA.

[52] Korte A., Myers J., Shih N.Y., Egan R.W., Clark M.A. (1990). Characterization and tissue distribution of H3 histamine receptors in guinea pigs by N alpha-methylhistamine. Biochem. Biophys. Res. Commun..

[53] van Rijn R.M., van Marle A., Chazot P.L., Langemeijer E., Qin Y., Shenton F.C., Lim H.D., Zuiderveld O.P., Sansuk K., Dy M. (2008). Cloning and characterization of dominant negative splice variants of the human histamine H4 receptor. Biochem. J..

[54] Pillot C., Heron A., Cochois V., Tardivel-Lacombe J., Ligneau X., Schwartz J.C., Arrang J.M. (2002). A detailed mapping of the histamine H(3) receptor and its gene transcripts in rat brain. Neuroscience.

[55] Shan L., Bossers K., Unmehopa U., Bao A.M., Swaab D.F. (2012). Alterations in the histaminergic system in Alzheimer’s disease: A postmortem study. Neurobiol. Aging.

[56] Jin C.Y., Anichtchik O., Panula P. (2009). Altered histamine H3 receptor radioligand binding in post-mortem brain samples from subjects with psychiatric diseases. Br. J. Pharmacol..

[57] Poveda R., Fernandez-Duenas V., Fernandez A., Sanchez S., Puig M.M., Planas E. (2006). Synergistic interaction between fentanyl and the histamine H3 receptor agonist R-(alpha)-methylhistamine, on the inhibition of nociception and plasma extravasation in mice. Eur. J. Pharmacol..

[58] Schlicker E., Kathmann M. (2017). Role of the Histamine H(3) Receptor in the Central Nervous System. Handb. Exp. Pharmacol..

[59] Arrang J.M., Garbarg M., Lancelot J.C., Lecomte J.M., Pollard H., Robba M., Schunack W., Schwartz J.C. (1987). Highly potent and selective ligands for histamine H3-receptors. Nature.

[60] Pollard H., Moreau J., Arrang J.M., Schwartz J.C. (1993). A detailed autoradiographic mapping of histamine H3 receptors in rat brain areas. Neuroscience.

[61] Jansen F.P., Mochizuki T., Maeyama K., Leurs R., Timmerman H. (2000). Characterization of histamine H3 receptors in mouse brain using the H3 antagonist [125I]iodophenpropit. Naunyn Schmiedebergs Arch. Pharmacol..

[62] Martinez-Mir M.I., Pollard H., Moreau J., Arrang J.M., Ruat M., Traiffort E., Schwartz J.C., Palacios J.M. (1990). Three histamine receptors (H1, H2 and H3) visualized in the brain of human and non-human primates. Brain Res..

[63] Chazot P.L., Hann V., Wilson C., Lees G., Thompson C.L. (2001). Immunological identification of the mammalian H3 histamine receptor in the mouse brain. Neuroreport.

[64] González-Sepúlveda M., Rosell S., Hoffmann H.M., Castillo-Ruiz M.d.M., Mignon V., Moreno-Delgado D., Vignes M., Díaz J., Sabriá J., Ortiz J. (2013). Cellular distribution of the histamine H3 receptor in the basal ganglia: Functional modulation of dopamine and glutamate neurotransmission. Basal Ganglia.

[65] Aquino-Miranda G., Escamilla-Sanchez J., Gonzalez-Pantoja R., Bueno-Nava A., Arias-Montano J.A. (2016). Histamine H3 receptor activation inhibits dopamine synthesis but not release or uptake in rat nucleus accumbens. Neuropharmacology.

[66] Shan L., Bossers K., Luchetti S., Balesar R., Lethbridge N., Chazot P.L., Bao A.M., Swaab D.F. (2012). Alterations in the histaminergic system in the substantia nigra and striatum of Parkinson’s patients: A postmortem study. Neurobiol. Aging.

[67] Davies S., Lujan K.S., Rappaport E.J., Valenzuela C.F., Savage D.D. (2023). Effect of moderate prenatal ethanol exposure on the differential expression of two histamine H3 receptor isoforms in different brain regions of adult rat offspring. Front. Neurosci..

[68] Clark E.A., Hill S.J. (1996). Sensitivity of histamine H3 receptor agonist-stimulated [35S]GTP gamma[S] binding to pertussis toxin. Eur. J. Pharmacol..

[69] Inoue A., Raimondi F., Kadji F.M.N., Singh G., Kishi T., Uwamizu A., Ono Y., Shinjo Y., Ishida S., Arang N. (2019). Illuminating G-Protein-Coupling Selectivity of GPCRs. Cell.

[70] Schihada H., Shekhani R., Schulte G. (2021). Quantitative assessment of constitutive G protein-coupled receptor activity with BRET-based G protein biosensors. Sci. Signal.

[71] Rouleau A., Ligneau X., Tardivel-Lacombe J., Morisset S., Gbahou F., Schwartz J.C., Arrang J.M. (2002). Histamine H3-receptor-mediated [35S]GTP gamma[S] binding: Evidence for constitutive activity of the recombinant and native rat and human H3 receptors. Br. J. Pharmacol..

[72] Sallmen T., Lozada A.F., Anichtchik O.V., Beckman A.L., Leurs R., Panula P. (2003). Changes in hippocampal histamine receptors across the hibernation cycle in ground squirrels. Hippocampus.

[73] Schihada H., Ma X., Zabel U., Vischer H.F., Schulte G., Leurs R., Pockes S., Lohse M.J. (2020). Development of a Conformational Histamine H(3) Receptor Biosensor for the Synchronous Screening of Agonists and Inverse Agonists. ACS Sens..

[74] Gao M., Dekker M.E., Leurs R., Vischer H.F. (2024). Pharmacological characterization of seven human histamine H(3) receptor isoforms. Eur. J. Pharmacol..

[75] Rahman S.N., Imhaouran F., Leurs R., Christopoulos A., Valant C., Langmead C.J. (2023). Ligand-directed biased agonism at human histamine H(3) receptor isoforms across Galpha(i/o)- and beta-arrestin2-mediated pathways. Biochem. Pharmacol..

[76] Rahman S.N., McNaught-Flores D.A., Huppelschoten Y., da Costa Pereira D., Christopoulos A., Leurs R., Langmead C.J. (2023). Structural and Molecular Determinants for Isoform Bias at Human Histamine H(3) Receptor Isoforms. ACS Chem. Neurosci..

[77] Staus D.P., Strachan R.T., Manglik A., Pani B., Kahsai A.W., Kim T.H., Wingler L.M., Ahn S., Chatterjee A., Masoudi A. (2016). Allosteric nanobodies reveal the dynamic range and diverse mechanisms of G-protein-coupled receptor activation. Nature.

[78] Sahlholm K., Nilsson J., Marcellino D., Fuxe K., Arhem P. (2012). Voltage sensitivities and deactivation kinetics of histamine H(3) and H(4) receptors. Biochim. Biophys. Acta.

[79] Shen Q., Tang X., Wen X., Cheng S., Xiao P., Zang S.K., Shen D.D., Jiang L., Zheng Y., Zhang H. (2024). Molecular Determinant Underlying Selective Coupling of Primary G-Protein by Class A GPCRs. Adv. Sci..

[80] Ballesteros J.A., Jensen A.D., Liapakis G., Rasmussen S.G., Shi L., Gether U., Javitch J.A. (2001). Activation of the beta 2-adrenergic receptor involves disruption of an ionic lock between the cytoplasmic ends of transmembrane segments 3 and 6. J. Biol. Chem..

[81] Ma X., Segura M.A., Zarzycka B., Vischer H.F., Leurs R. (2021). Analysis of Missense Variants in the Human Histamine Receptor Family Reveals Increased Constitutive Activity of E410(6.30x30)K Variant in the Histamine H(1) Receptor. Int. J. Mol. Sci..

[82] Jumper J., Evans R., Pritzel A., Green T., Figurnov M., Ronneberger O., Tunyasuvunakool K., Bates R., Zidek A., Potapenko A. (2021). Highly accurate protein structure prediction with AlphaFold. Nature.

[83] Betke K.M., Wells C.A., Hamm H.E. (2012). GPCR mediated regulation of synaptic transmission. Prog. Neurobiol..

[84] Zurawski Z., Yim Y.Y., Alford S., Hamm H.E. (2019). The expanding roles and mechanisms of G protein-mediated presynaptic inhibition. J. Biol. Chem..

[85] Proft J., Weiss N. (2015). G protein regulation of neuronal calcium channels: Back to the future. Mol. Pharmacol..

[86] Dolphin A.C., Lee A. (2020). Presynaptic calcium channels: Specialized control of synaptic neurotransmitter release. Nat. Rev. Neurosci..

[87] Takeshita Y., Watanabe T., Sakata T., Munakata M., Ishibashi H., Akaike N. (1998). Histamine modulates high-voltage-activated calcium channels in neurons dissociated from the rat tuberomammillary nucleus. Neuroscience.

[88] Dong H., Li M., Yan Y., Qian T., Lin Y., Ma X., Vischer H.F., Liu C., Li G., Wang H. (2023). Genetically encoded sensors for measuring histamine release both in vitro and in vivo. Neuron.

[89] Morrey C., Estephan R., Abbott G.W., Levi R. (2008). Cardioprotective effect of histamine H_3_-receptor activation: Pivotal role of G beta gamma-dependent inhibition of voltage-operated Ca^2+^ channels. J. Pharmacol. Exp. Ther..

[90] Seyedi N., Mackins C.J., Machida T., Reid A.C., Silver R.B., Levi R. (2005). Histamine H3-receptor-induced attenuation of norepinephrine exocytosis: A decreased protein kinase a activity mediates a reduction in intracellular calcium. J. Pharmacol. Exp. Ther..

[91] Jeremic D., Sanchez-Rodriguez I., Jimenez-Diaz L., Navarro-Lopez J.D. (2021). Therapeutic potential of targeting G protein-gated inwardly rectifying potassium (GIRK) channels in the central nervous system. Pharmacol. Ther..

[92] Vázquez-Vázquez H., Gonzalez-Sandoval C., Vega A.V., Arias-Montaño J.-A., Barral J. (2020). Histamine H3 Receptor Activation Modulates Glutamate Release in the Corticostriatal Synapse by Acting at CaV2.1 (P/Q-Type) Calcium Channels and GIRK (KIR3) Potassium Channels. Cell. Mol. Neurobiol..

[93] Levi R., Seyedi N., Schaefer U., Estephan R., Mackins C.J., Tyler E., Silver R.B. (2007). Histamine H3-receptor signaling in cardiac sympathetic nerves: Identification of a novel MAPK-PLA2-COX-PGE2-EP3R pathway. Biochem. Pharmacol..

[94] Starke K., Gothert M., Kilbinger H. (1989). Modulation of neurotransmitter release by presynaptic autoreceptors. Physiol. Rev..

[95] Westerink B.H., Cremers T.I., De Vries J.B., Liefers H., Tran N., De Boer P. (2002). Evidence for activation of histamine H3 autoreceptors during handling stress in the prefrontal cortex of the rat. Synapse.

[96] Li M., Hu J., Chen Z., Meng J., Wang H., Ma X., Luo X. (2006). Evidence for histamine as a neurotransmitter in the cardiac sympathetic nervous system. Am. J. Physiol. Heart Circ. Physiol..

[97] Sundvik M., Kudo H., Toivonen P., Rozov S., Chen Y.C., Panula P. (2011). The histaminergic system regulates wakefulness and orexin/hypocretin neuron development via histamine receptor H1 in zebrafish. FASEB J..

[98] Alves-Rodrigues A., Timmerman H., Willems E., Lemstra S., Zuiderveld O.P., Leurs R. (1998). Pharmacological characterisation of the histamine H3 receptor in the rat hippocampus. Brain Res..

[99] Fuder H., Muscholl E. (1995). Heteroreceptor-mediated modulation of noradrenaline and acetylcholine release from peripheral nerves. Rev. Physiol. Biochem. Pharmacol..

[100] Schlicker E., Werthwein S., Zentner J. (1999). Histamine H3 receptor-mediated inhibition of noradrenaline release in the human brain. Fundam. Clin. Pharmacol..

[101] Schlicker E., Malinowska B., Kathmann M., Gothert M. (1994). Modulation of neurotransmitter release via histamine H3 heteroreceptors. Fundam. Clin. Pharmacol..

[102] Schlicker E., Behling A., Lummen G., Gothert M. (1992). Histamine H3A receptor-mediated inhibition of noradrenaline release in the mouse brain cortex. Naunyn Schmiedebergs Arch. Pharmacol..

[103] Schlicker E., Fink K., Hinterthaner M., Gothert M. (1989). Inhibition of noradrenaline release in the rat brain cortex via presynaptic H3 receptors. Naunyn Schmiedebergs Arch. Pharmacol..

[104] Smits R.P., Mulder A.H. (1991). Inhibitory effects of histamine on the release of serotonin and noradrenaline from rat brain slices. Neurochem. Int..

[105] Ishikawa S., Sperelakis N. (1987). A novel class (H3) of histamine receptors on perivascular nerve terminals. Nature.

[106] Schlicker E., Betz R., Gothert M. (1988). Histamine H3 receptor-mediated inhibition of serotonin release in the rat brain cortex. Naunyn Schmiedebergs Arch. Pharmacol..

[107] Fink K., Schlicker E., Neise A., Gothert M. (1990). Involvement of presynaptic H3 receptors in the inhibitory effect of histamine on serotonin release in the rat brain cortex. Naunyn Schmiedebergs Arch. Pharmacol..

[108] Schlicker E., Fink K., Detzner M., Gothert M. (1993). Histamine inhibits dopamine release in the mouse striatum via presynaptic H3 receptors. J. Neural Transm. Gen. Sect..

[109] Arias-Montano J.A., Floran B., Garcia M., Aceves J., Young J.M. (2001). Histamine H(3) receptor-mediated inhibition of depolarization-induced, dopamine D(1) receptor-dependent release of [(3)H]-gamma-aminobutryic acid from rat striatal slices. Br. J. Pharmacol..

[110] Matsubara T., Moskowitz M.A., Huang Z. (1992). UK-14,304, R(-)-alpha-methyl-histamine and SMS 201-995 block plasma protein leakage within dura mater by prejunctional mechanisms. Eur. J. Pharmacol..

[111] Dimitriadou V., Rouleau A., Trung Tuong M.D., Newlands G.J., Miller H.R., Luffau G., Schwartz J.C., Garbarg M. (1997). Functional relationships between sensory nerve fibers and mast cells of dura mater in normal and inflammatory conditions. Neuroscience.

[112] Gomez-Ramirez J., Ortiz J., Blanco I. (2002). Presynaptic H3 autoreceptors modulate histamine synthesis through cAMP pathway. Mol. Pharmacol..

[113] Torrent A., Moreno-Delgado D., Gomez-Ramirez J., Rodriguez-Agudo D., Rodriguez-Caso C., Sanchez-Jimenez F., Blanco I., Ortiz J. (2005). H3 autoreceptors modulate histamine synthesis through calcium/calmodulin- and cAMP-dependent protein kinase pathways. Mol. Pharmacol..

[114] Gbahou F., Rouleau A., Arrang J.M. (2012). The histamine autoreceptor is a short isoform of the H(3) receptor. Br. J. Pharmacol..

[115] Parks G.S., Olivas N.D., Ikrar T., Sanathara N.M., Wang L., Wang Z., Civelli O., Xu X. (2014). Histamine inhibits the melanin-concentrating hormone system: Implications for sleep and arousal. J. Physiol..

[116] Zhou F.W., Xu J.J., Zhao Y., LeDoux M.S., Zhou F.M. (2006). Opposite functions of histamine H1 and H2 receptors and H3 receptor in substantia nigra pars reticulata. J. Neurophysiol..

[117] Lundius E.G., Sanchez-Alavez M., Ghochani Y., Klaus J., Tabarean I.V. (2010). Histamine influences body temperature by acting at H1 and H3 receptors on distinct populations of preoptic neurons. J. Neurosci..

[118] Sethi J., Sanchez-Alavez M., Tabarean I.V. (2011). Kv4.2 mediates histamine modulation of preoptic neuron activity and body temperature. PLoS ONE.

[119] Zheng Y., Fan L., Fang Z., Liu Z., Chen J., Zhang X., Wang Y., Zhang Y., Jiang L., Chen Z. (2023). Postsynaptic histamine H3 receptors in ventral basal forebrain cholinergic neurons modulate contextual fear memory. Cell Rep..

[120] Wang N., Ma J., Liu J., Wang J., Liu C., Wang H., Liu Y., Yan H., Jiang S. (2019). Histamine H3 Receptor Antagonist Enhances Neurogenesis and Improves Chronic Cerebral Hypoperfusion-Induced Cognitive Impairments. Front. Pharmacol..

[121] Lai X., Ye L., Liao Y., Jin L., Ma Q., Lu B., Sun Y., Shi Y., Zhou N. (2016). Agonist-induced activation of histamine H3 receptor signals to extracellular signal-regulated kinases 1 and 2 through PKC-, PLD-, and EGFR-dependent mechanisms. J. Neurochem..

[122] Mariottini C., Scartabelli T., Bongers G., Arrigucci S., Nosi D., Leurs R., Chiarugi A., Blandina P., Pellegrini-Giampietro D.E., Passani M.B. (2009). Activation of the histaminergic H3 receptor induces phosphorylation of the Akt/GSK-3 beta pathway in cultured cortical neurons and protects against neurotoxic insults. J. Neurochem..

[123] Bongers G., Sallmen T., Passani M.B., Mariottini C., Wendelin D., Lozada A., Marle A., Navis M., Blandina P., Bakker R.A. (2007). The Akt/GSK-3beta axis as a new signaling pathway of the histamine H(3) receptor. J. Neurochem..

[124] Molina-Hernandez A., Nunez A., Arias-Montano J.A. (2000). Histamine H3-receptor activation inhibits dopamine synthesis in rat striatum. Neuroreport.

[125] Molina-Hernandez A., Nunez A., Sierra J.J., Arias-Montano J.A. (2001). Histamine H3 receptor activation inhibits glutamate release from rat striatal synaptosomes. Neuropharmacology.

[126] Moreno E., Hoffmann H., Gonzalez-Sepulveda M., Navarro G., Casado V., Cortes A., Mallol J., Vignes M., McCormick P.J., Canela E.I. (2011). Dopamine D1-histamine H3 receptor heteromers provide a selective link to MAPK signaling in GABAergic neurons of the direct striatal pathway. J. Biol. Chem..

[127] Xu J., Pittenger C. (2023). The histamine H3 receptor modulates dopamine D2 receptor-dependent signaling pathways and mouse behaviors. J. Biol. Chem..

[128] Moreno E., Moreno-Delgado D., Navarro G., Hoffmann H.M., Fuentes S., Rosell-Vilar S., Gasperini P., Rodriguez-Ruiz M., Medrano M., Mallol J. (2014). Cocaine disrupts histamine H3 receptor modulation of dopamine D1 receptor signaling: Sigma1-D1-H3 receptor complexes as key targets for reducing cocaine’s effects. J. Neurosci..

[129] Moreno-Delgado D., Puigdellivol M., Moreno E., Rodriguez-Ruiz M., Botta J., Gasperini P., Chiarlone A., Howell L.A., Scarselli M., Casado V. (2020). Modulation of dopamine D(1) receptors via histamine H(3) receptors is a novel therapeutic target for Huntington’s disease. Elife.

[130] Ferrada C., Ferre S., Casado V., Cortes A., Justinova Z., Barnes C., Canela E.I., Goldberg S.R., Leurs R., Lluis C. (2008). Interactions between histamine H3 and dopamine D2 receptors and the implications for striatal function. Neuropharmacology.

[131] Ferrada C., Moreno E., Casado V., Bongers G., Cortes A., Mallol J., Canela E.I., Leurs R., Ferre S., Lluis C. (2009). Marked changes in signal transduction upon heteromerization of dopamine D1 and histamine H3 receptors. Br. J. Pharmacol..

[132] Dale N.C., Johnstone E.K.M., Pfleger K.D.G. (2022). GPCR heteromers: An overview of their classification, function and physiological relevance. Front. Endocrinol..

[133] Ferre S., Casado V., Devi L.A., Filizola M., Jockers R., Lohse M.J., Milligan G., Pin J.P., Guitart X. (2014). G protein-coupled receptor oligomerization revisited: Functional and pharmacological perspectives. Pharmacol. Rev..

[134] Rodriguez-Ruiz M., Moreno E., Moreno-Delgado D., Navarro G., Mallol J., Cortes A., Lluis C., Canela E.I., Casado V., McCormick P.J. (2017). Heteroreceptor Complexes Formed by Dopamine D(1), Histamine H(3), and N-Methyl-D-Aspartate Glutamate Receptors as Targets to Prevent Neuronal Death in Alzheimer’s Disease. Mol. Neurobiol..

[135] Marquez-Gomez R., Robins M.T., Gutierrez-Rodelo C., Arias J.M., Olivares-Reyes J.A., van Rijn R.M., Arias-Montano J.A. (2018). Functional histamine H(3) and adenosine A(2A) receptor heteromers in recombinant cells and rat striatum. Pharmacol. Res..

[136] Morales-Figueroa G.E., Rivera-Ramirez N., Gonzalez-Pantoja R., Escamilla-Sanchez J., Garcia-Hernandez U., Galvan E.J., Arias-Montano J.A. (2019). Adenosine A(2A) and histamine H(3) receptors interact at the cAMP/PKA pathway to modulate depolarization-evoked [(3)H]-GABA release from rat striato-pallidal terminals. Purinergic Signal..

[137] El Khamlichi C., Cobret L., Arrang J.M., Morisset-Lopez S. (2021). BRET Analysis of GPCR Dimers in Neurons and Non-Neuronal Cells: Evidence for Inactive, Agonist, and Constitutive Conformations. Int. J. Mol. Sci..

[138] Coge F., Guenin S.P., Renouard-Try A., Rique H., Ouvry C., Fabry N., Beauverger P., Nicolas J.P., Galizzi J.P., Boutin J.A. (1999). Truncated isoforms inhibit [3H]prazosin binding and cellular trafficking of native human alpha1A-adrenoceptors. Biochem. J..

[139] Wess J., Oteng A.B., Rivera-Gonzalez O., Gurevich E.V., Gurevich V.V. (2023). beta-Arrestins: Structure, Function, Physiology, and Pharmacological Perspectives. Pharmacol. Rev..

[140] Underwood O., Haider R.S., Sanchez J., Canals M. (2024). Arrestin-centred interactions at the membrane and their conformational determinants. Br. J. Pharmacol..

[141] Perez-Garcia C., Morales L., Alguacil L.F. (1998). Histamine H3 receptor desensitization in the guinea-pig ileum. Eur. J. Pharmacol..

[142] Garduno-Torres B., Arias-Montano J.A. (2006). Homologous down-regulation of histamine H3 receptors in rat striatal slices. Synapse.

[143] Osorio-Espinoza A., Escamilla-Sanchez J., Aquino-Jarquin G., Arias-Montano J.A. (2014). Homologous desensitization of human histamine H(3) receptors expressed in CHO-K1 cells. Neuropharmacology.

[144] Garcia-Galvez A.M., Escamilla-Sanchez J., Flores-Maldonado C., Contreras R.G., Arias J.M., Arias-Montano J.A. (2018). Differential homologous desensitization of the human histamine H(3) receptors of 445 and 365 amino acids expressed in CHO-K1 cells. Neurochem. Int..

[145] Montejo-Lopez W., Rivera-Ramirez N., Escamilla-Sanchez J., Garcia-Hernandez U., Arias-Montano J.A. (2016). Heterologous, PKC-Mediated Desensitization of Human Histamine H3 Receptors Expressed in CHO-K1 Cells. Neurochem. Res..

[146] Riddy D.M., Cook A.E., Diepenhorst N.A., Bosnyak S., Brady R., Mannoury la Cour C., Mocaer E., Summers R.J., Charman W.N., Sexton P.M. (2017). Isoform-Specific Biased Agonism of Histamine H3 Receptor Agonists. Mol. Pharmacol..

[147] Giros B., Sokoloff P., Martres M.P., Riou J.F., Emorine L.J., Schwartz J.C. (1989). Alternative splicing directs the expression of two D2 dopamine receptor isoforms. Nature.

[148] Liu X.Y., Liu Z.C., Sun Y.G., Ross M., Kim S., Tsai F.F., Li Q.F., Jeffry J., Kim J.Y., Loh H.H. (2011). Unidirectional cross-activation of GRPR by MOR1D uncouples itch and analgesia induced by opioids. Cell.

[149] Chakrabarti S., Liu N.J., Gintzler A.R. (2021). Relevance of Mu-Opioid Receptor Splice Variants and Plasticity of Their Signaling Sequelae to Opioid Analgesic Tolerance. Cell Mol. Neurobiol..

[150] Piltonen M., Parisien M., Gregoire S., Chabot-Dore A.J., Jafarnejad S.M., Berube P., Djambazian H., Sladek R., Geneau G., Willett P. (2019). Alternative Splicing of the Delta-Opioid Receptor Gene Suggests Existence of New Functional Isoforms. Mol. Neurobiol..

